# Meditation expertise influences response bias and prestimulus alpha activity in the somatosensory signal detection task

**DOI:** 10.1111/psyp.14712

**Published:** 2024-11-18

**Authors:** Maik Mylius, Simon Guendelman, Fivos Iliopoulos, Vittorio Gallese, Laura Kaltwasser

**Affiliations:** ^1^ Berlin School of Mind and Brain Humboldt‐Universität zu Berlin Berlin Germany; ^2^ Institute of Computer Science Georg‐August‐Universität Göttingen Göttingen Germany; ^3^ Department of Neurology Max Planck Institute for Human Cognitive and Brain Sciences Leipzig Germany; ^4^ Unit of Neuroscience, Department of Medicine and Surgery University of Parma Parma Italy

**Keywords:** alpha modulation, interoception, meditation, mindfulness, prestimulus alpha, somatosensory awareness, somatosensory signal detection, Vipassana

## Abstract

This study investigates the proposed mechanism of mindfulness, its impact on body awareness and interoception, and its potential benefits for mental and physical health. Using psychophysical assessments, we compared 31 expert meditators with 33 matched controls (non‐meditators who engage in regular reading, more than 5 h per week) in terms of somatosensory accuracy with a somatosensory signal detection task (SSDT) and interoceptive sensibility via self‐report measures. We hypothesized that meditators would demonstrate superior somatosensory accuracy, indicative of heightened body awareness, potentially linked to increased alpha modulation in the somatosensory cortex, as observed via electroencephalography (EEG). In the SSDT, participants attempted to detect near‐threshold tactile stimuli presented with a non‐informative light in half of the trials. Contrary to our expectations, the findings showed that meditators had a lower decision threshold rather than higher accuracy. EEG results corroborated earlier research, indicating reduced prestimulus alpha power in meditators, suggesting enhanced alpha modulation. Furthermore, a trial‐by‐trial analysis revealed a negative correlation between prestimulus alpha activity and tactile perception. Compared to controls, meditators also reported greater interoceptive sensibility, less emotional suppression, and fewer difficulties in describing feelings. These findings may imply that enhanced tactile perception is associated with lower prestimulus alpha activity by reducing sensory filtering in the somatosensory cortex, thus increasing response rates without necessarily improving accuracy among meditators.

## Abstract 2

Our findings add to the current literature on mindfulness meditation and body awareness by assessing somatosensory perception combined with an EEG prestimulus alpha investigation in meditators. We found higher interoceptive sensibility in the mediation group and supplement previous evidence by linking prestimulus alpha activity to behavioral performance and finding lower prestimulus alpha activity in meditators, which is potentially lowering the somatosensory decision criterion in the meditation group.

## 1 INTRODUCTION

Mindfulness is described as non‐judgmental awareness of present‐moment experience (Kabat‐Zinn, 2005). The practice of mindfulness meditation has been shown to have positive effects on well‐being and both physical and psychological health (Goldberg, Riordan, et al., 2022; van Agteren et al., 2021). One central type of mindfulness practice is body‐scan meditation, and recent evidence indicates its effectiveness in reducing stress (Schultchen et al., 2019), insomnia (Bruin et al., 2020), and depression (Corbett et al., 2019), as well as its positive effects on life satisfaction (Dambrun et al., 2019; Kropp & Sedlmeier, 2019). However, the mechanisms by which this non‐judgmental attention interacts with body sensations to relieve such symptoms remain unclear. The practice of body‐centered mindfulness meditation involves focusing on present‐moment bodily sensations, emphasizing the experience of these sensations as they are, without bias or affective responding (Farb et al., 2015). Owing to the predominant focus on internal signals during mindfulness meditation, one way understand its benefits is through “embodied emotion regulation” (Guendelman et al., 2017) via altered interoception—the “receiving, accessing and appraising of internal bodily signals” (Farb et al., 2015)—and the closely related construct of body awareness (Hölzel et al., 2011). Various neuroimaging evidence points in this direction, demonstrating augmented gray matter density and heightened activation within the anterior insula among mindfulness meditators, an area that has been described as the central hub of interoception (Fox et al., 2014; Fox et al., 2016; Lazar et al., 2005).

### 1.1 Mindfulness and body awareness

After a mindfulness intervention, participants generally score higher on subjective scales of body awareness, known as interoceptive sensibility (Bornemann et al., 2014; D'Antoni et al., 2022; Mehling et al., 2012). However, evidence for increased interoceptive accuracy measured via objective behavioral tasks, such as the commonly used heartbeat perception task, is contradictory. Although a large‐sample intervention study revealed a positive relationship between meditation practice and accuracy in heartbeat perception (Bornemann & Singer, 2017), overwhelming evidence has not shown a relationship between heartbeat perception and meditation (Khalsa et al., 2019; Treves et al., 2019).

Nevertheless, other objective measures were able to establish differences in body awareness between long‐term meditators (LTMs) and non‐meditators. Fox et al. (2012) reported that long‐term meditators with more lifetime practice also exhibit greater coherence between the subjective clarity of body regions, two‐point discriminatory thresholds, and cortical representations within the primary somatosensory cortex. Kerr et al. (2008) found that tactile acuity was greater in regular Thai‐Chi practitioners than in a random control group, indicating increased somatosensory awareness in mind–body meditation practitioners. Sze et al. (2010) reported greater coherence between subjective ratings and cardiac indicators of emotions during emotion‐eliciting films in long‐term meditators than in dancers and controls. In a small intervention study, Mirams et al. (2013) reported reduced tactile misperception in meditators via a somatosensory signal detection task (SSDT). Taken together, these findings imply a relationship between meditation practice and objective measures of body awareness, as confirmed for mindfulness interventions and expert meditators by a recent meta‐analysis (Treves et al., 2019).

### 1.2 Somatosensory signal detection task

Signal detection theory is a psychophysical framework used to understand how weak or ambiguous sensory stimuli are detected. The theory posits two primary constructs: sensitivity, also referred to as accuracy, and criterion. Both are calculated based on the hit rate (stimulus‐present, report‐yes) and false alarm rate (stimulus‐absent, report‐yes). The term “sensitivity” is used to describe the ability to correctly identify the presence or absence of a stimulus. In contrast, the term ‘criterion’ is used to describe the decision threshold for a response. Individuals who exhibit higher levels of sensitivity are more likely to accurately detect or reject stimuli. Conversely, those with lower criterion are more likely to report sensations, even when the stimulus is weak or ambiguous.

The somatosensory signal detection task (SSDT) uses a signal detection paradigm in which near‐threshold vibrotactile stimuli are delivered to the left index finger. However, to measure the robustness of somatosensory processing against visual stimuli, tactile stimuli are paired with light flashes in 50% of the trials (Lloyd et al., 2008) (for the trial scheme, also see Figure 2a). The results showed that the light flash was able to elicit an illusory touch sensation in some cases. Positive correlations between the false alarm rate, reported somatisation and physical symptoms were found, indicating that the SSDT can capture somatosensory disturbances (Brown et al., 2010, 2012). Furthermore, the false alarm rate appears to be a robust measure over time (McKenzie et al., 2010).

By measuring sensitivity and decision criterion, the SSDT can provide insights into the mechanisms underlying somatosensory awareness in expert meditators and may also be useful for identifying somatosensory disturbances related to specific physical or mental health conditions (Brown et al., 2012). By implementing the SSDT in an interventional study, Mirams et al. (2013) reported that sensitivity (d′) increased and false alarms decreased after a seven‐day body‐scan meditation intervention compared with a story‐listening control group. In addition to assessing the transient effects of meditative practice on sensitivity in interventional design studies, it is expected that long‐term meditation experts would also demonstrate this effect in cross‐sectional studies, especially in traditions that emphasize body‐centered meditation.

### 1.3 Alpha oscillations

Alpha band frequency oscillations (8–13 Hz) play a complex role in somatosensory perception and have been associated with various cognitive functions, including attention, arousal, and sensory processing. Research suggests that alpha activity may play a modulatory role in somatosensory perception by influencing sensory gating, the ability of the brain to filter out irrelevant sensory information while selectively attending to important stimuli (Jensen & Mazaheri, 2010; Weisz et al., 2014); cortical excitability, allowing or preventing early sensory responses to reach consciousness (Jensen & Mazaheri, 2010; Klimesch et al., 2007); and attentional mechanisms. Higher levels of alpha activity may reflect the inhibition of task‐irrelevant sensory processing, allowing individuals to focus on relevant somatosensory information more effectively (Forschack et al., 2017; Foxe & Snyder, 2011; Haegens et al., 2011; Jones et al., 2010; van Ede et al., 2011; Whitmarsh et al., 2014).

Regardless of the underlying mechanism of interaction, low levels of alpha before stimulation have frequently been associated with improvements in task performance in tactile discrimination and detection (Baumgarten et al., 2016; Jones et al., 2010; van Ede et al., 2011), although other groups have reported a quadratic relationship between prestimulus alpha and performance, with the highest hit rates corresponding to intermediate alpha power levels (Ai & Ro, 2014; Linkenkaer‐Hansen et al., 2004; Weisz et al., 2014; Zhang & Ding, 2010). When false reports were additionally assessed, decreased alpha oscillations were found to increase false alarms. Therefore, in terms of signal detection, decreased alpha oscillations did not increase the sensitivity but rather lowered the decision criterion (Craddock et al., 2017, 2019).

Studies investigating the influence of alpha power on perceptual decision‐making usually relate absolute prestimulus alpha power values to behavioral outcomes (Baumgarten et al., 2016; Craddock et al., 2017; Forschack et al., 2017; Weisz et al., 2014). When investigating shifts in alpha power in response to an attentional cue, Jones et al. (2010), Kerr et al. (2011), and Haegens et al. (2012) used a relative measure of alpha activity, comparing prestimulus alpha activity to a baseline during the onset of the attentional cue. Hence, it is here referred to as baselined or baseline‐corrected alpha activity and represents the modulation of alpha activity after the cue.

Kerr et al. (2011) compared the ability to modulate alpha activity over the somatosensory cortex between a meditation intervention group and a control group using a cued attention modulation task. Greater alpha modulation, i.e., higher differences in baselined alpha power, was found in meditators than in controls when attending hand vs. foot trials were compared. The finding that meditation increases attentional alpha modulation within the body may implicate greater attentional control in body perception, which may be one potential interoceptive mechanism for the salutary effects of mindfulness (Kerr et al., 2013).

Craddock et al. (2017) used the aforementioned SSDT to investigate the influence of prestimulus alpha activity on response rate on a trial‐by‐trial basis. The findings revealed that lower prestimulus alpha activity over the somatosensory cortex predicts a higher likelihood of reporting a touch sensation during the SSDT, i.e., a lower criterion but not higher sensitivity, as previously assumed due to its relation to attention (Jones et al., 2010; Samaha et al., 2020; Weisz et al., 2014). Given that meditators show greater alpha modulation over somatosensory areas (Kerr et al., 2011) and assuming that there is similar alpha activity at cue‐baseline, one would expect prestimulus alpha over attended regions to be lower in meditators. However, considering that lower prestimulus alpha is related to a lower decision criterion in signal detection (Craddock et al., 2017), it remains unclear whether alpha activity improves task accuracy or just influences reportability, i.e., whether meditators would also exhibit an increase in sensitivity in the SSDT, as in Mirams et al. (2013), or just a lowering of the criterion.

### 1.4 Present study and experimental hypotheses

In the present study, the primary aim was to investigate the trait effects of regular meditation practice, with a focus on somatosensory perception. In our experimental design, we compared a group of regular meditators with a practice on body sensations to a matched group of regular readers as controls. Using the SSDT while simultaneously recording EEG alpha activity and assessing interoceptive sensibility, mindfulness, physical symptoms, and emotional suppression with self‐report questionnaires, we investigated differences in somatosensory processing and body awareness in general. In this cross‐sectional study, we hypothesized that meditation experts would exhibit greater sensitivity in the SSDT in comparison to a group of non‐meditators, as in Mirams et al., 2013. Specifically, by controlling for prestimulus alpha activity over the primary somatosensory cortex (absolute and baseline‐corrected), we investigated the difference in alpha activity between the two groups and its effect on electro‐tactile detection performance. Based on previous findings, we expected that baselined alpha activity would be lower over the somatosensory cortex in the meditation group as a result of increased alpha modulation (Kerr et al., 2011). Additionally, we expected higher interoceptive sensibility, higher trait mindfulness, lower somatic symptoms, decreased emotional suppression, and lower alexithymia scores in the meditator group according to self‐report measures. Moreover, as an exploratory analysis, we investigated the differences in absolute somatosensory prestimulus alpha activity and the relationship between somatosensory prestimulus alpha activity and somatosensory signal detection with a trial‐by‐trial analysis. Furthermore, to examine the differences in alpha activity between groups without an a priori hypothesis about location, we conducted cluster‐based permutation tests with absolute and baseline‐corrected prestimulus alpha values. Finally, an exploratory correlation analysis between measures was performed, further corroborating the findings.

## 2 METHODS

### 2.1 Subjects

For our study, we recruited 31 meditation practitioners and 33 regular readers. Participants were required to engage in at least five hours of either meditation or reading literature per week and had been doing so for a minimum of two years. We selected an active control group of long‐term practicing readers with no meditation experience to prevent motivational effects from impacting task performance. Both reading and meditation involve prolonged periods of sustained attention without much movement, thus controlling for quiescence and extended periods of sustained attention. In the following, the participants of the reading group are referred to as “non‐meditators”.

Both groups were recruited separately, with advertisements stating the possibility of participating in an experiment investigating the influence of either reading or meditation on the brain. The reading group was recruited through flyers at the university, a recruitment database of the university for individuals interested in participating in psychological experiments, and announcements on other internet platforms (eBay Kleinanzeigen and Facebook groups). The inclusion criteria were assessed during a phone interview. Participants in the non‐meditator group reported no regular meditation or yoga practice or participation in a meditation retreat. Three non‐meditators reported meditation practice but remained in the group due to the low frequency of practice (less than 2 sessions of 20 min per week). The meditation group was recruited through announcements in Facebook groups for meditation and local meditation communities. The predominant meditation style was Vipassana (*n* = 28), of whom 24 participants practiced within the Goenka Vipassana tradition. Two participants practiced Zen meditation, and one participant practiced breath‐awareness meditation. The meditators reported an average of 5.92 years of meditation practice (*SD* = 5.44), with an average of 9.77 sessions (*SD* = 3.66) per week and 48.87 min of meditation per session (*SD* = 12.96), equating to an average of 8.09 h of weekly meditation practice (*SD* = 3.89) and an average total calculated meditation experience of 2628 h (*SD* = 3198). Six meditators unexpectedly reported meditating less than 5 h per week but were included in the group because they practiced at least five sessions per week for at least 30 min. Both groups were balanced for age (meditators range: 25–57 years, mean = 34.71, *SD* = 7.21 years; non‐meditators range: 21–46 years, mean = 31.52, *SD* = 7.22), *t*(62) = 1.76, *p* = .08, and gender (meditators: 12 women, 19 men; non‐meditators: 11 women, 22 men), *Χ* ^2^(1) = 0.20, *p* = .65. Participants were non‐smokers, right‐handed; were fluent English speakers; reported no current neurological, psychiatric, or cardiovascular conditions; reported no medication intake; and were of normal weight. The study was approved by the local ethics committee, and all the subjects provided written informed consent before participating. Participants were compensated with 10€/h or received course credit through the psychology research pool following participation.

### 2.2 Materials

#### 2.2.1 Questionnaires

The participants completed online questionnaires prior to the study. These questionnaires included demographic information such as sex, age, years of education, handedness, eyewear, smoking, medication intake, reading background, meditation background, and group activity. Additionally, the Mindfulness Attentional Awareness Scale (MAAS) (Brown & Ryan, 2003) was used to assess trait mindfulness, and the second version of the Multidimensional Assessment of Interoceptive Awareness (MAIA‐2) (Mehling et al., 2018) was used to assess interoceptive sensibility. Owing to a data entry error, a five‐item Likert scale was displayed instead of the official six‐item Likert scale. The option “Never” was thus coded as a 1 instead of a 0, whereas the value of “Always” remained at 5. The MAIA‐2 measures eight subfactors comprising “Noticing”, “Not‐Distracting”, “Not‐Worrying”, “Attention Regulation”, “Emotional Awareness”, “Self‐Regulation”, “Body Listening”, and “Trusting”. The Patient Health Questionnaire somatic symptom severity scale (PHQ‐15) (Kroenke et al., 2002) was used to evaluate somatisation symptoms such as headache or dizziness. The Toronto Alexithymia Scale (TAS) (Bagby et al., 1994) and the Emotion Regulation Questionnaire (ERQ) (Gross & John, 2003) were used to assess emotional awareness on the subscales of “Difficulty Describing Feelings”, “Difficulty Identifying Feelings”, and “Externally Oriented Thinking” (TAS) and emotion regulation on the subscales of “Expressive Suppression” and “Cognitive Reappraisal” (ERQ‐15). The Attentional Control Scale (ACS) (Derryberry & Reed, 2002) was assessed but not included in the analysis for the purpose of this paper. The questionnaire results were compared between groups with Welch *t*‐tests.

#### 2.2.2 Apparatus

During the experimental procedure, the participant was seated within a faradaic and acoustically shielded, light‐attenuated cabin and viewed the screen through a glass window. The participant received electrical stimulation via two ring electrodes placed on the left index finger and a constant current stimulator (DS‐5, Digitimer Ltd.) located outside the cabin. Electrical impulses were delivered through the DT‐8912 (Data Translation®) waveform generator. Additionally, a 5 mm red light‐emitting diode (LED) was fixed to the left index finger, as shown in Figure 2b. Cues and instructions were delivered via a stimulus computer with a 17‐inch display and a resolution of 1024 × 768 pixels, which was positioned approximately half a meter away from the participant behind the window. The procedure was programmed and conducted using MATLAB with the Psychophysics Toolbox extensions (Kleiner et al., 2007). The participants were able to respond via dedicated buttons on a computer keyboard.

### 2.3 Design and experimental procedure

**FIGURE 1:**
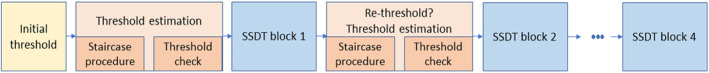
Experimental procedure. The experiment began with a preliminary manual estimation of the detection threshold. This was followed by an automated staircase procedure to estimate the 50% threshold, after which a threshold check was performed. If 4 to 6 out of 10 stimuli were detected, the experimental block commenced; otherwise, the staircase procedure was repeated. If a ceiling or floor effect was observed during the experimental block, the threshold estimation procedure was repeated to re‐establish the threshold.

The present study employed a between‐subjects design in which a group of long‐term meditators was compared to a group of non‐meditators using SSDT and EEG. Prior to the experiment, the participants completed online questionnaires and were then invited to attend the laboratory. After providing informed consent, EEG was placed, and ring electrodes and LED was placed on their left index finger. The experiential procedure then began with a thresholding estimation followed by the SSDT task, with the possibility of re‐thresholding (see Figure 1). The participants were left unaccompanied in the cabin during the experimental procedure, which also included a consecutive resting‐state recording and an emotion regulation task (the results of which will be reported elsewhere). Participants were subsequently compensated for their participation.

#### 2.3.1 Threshold acquisition

To estimate the 50% decision threshold, a 1‐up/1‐down adaptive staircase procedure was implemented to select intensity levels for the stimulations (Levitt, 1971). The validity of the resulting threshold was tested with 13 trials, of which 10 test trials had the estimated threshold intensity and three zero‐trials conveyed no current. If the participant responded with four to six hits for the 10 test trials, the threshold was granted as valid and used for the later SSDT (Lloyd et al., 2008). For more information on the thresholding procedure, see Supporting Information A1.

#### 2.3.2 Somatosensory signal detection task

**FIGURE 2:**
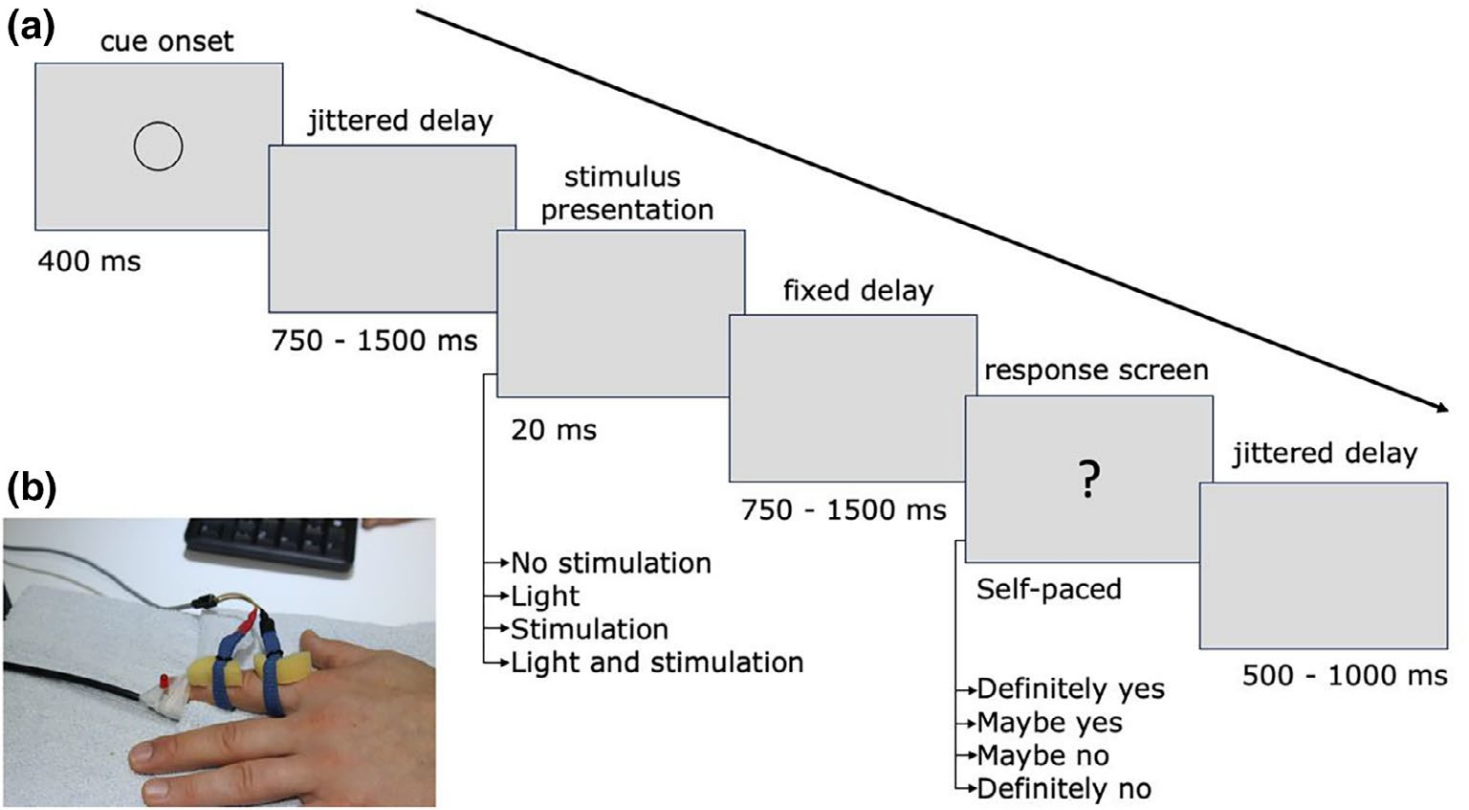
Trial sequence of the somatosensory signal detection task. (a) The trial begins with a visual cue followed by a blank screen. In the event period, one of the four stimulus conditions is delivered, followed by a fixed delay period before the onset of the response screen. The response screen features a question mark signaling the participant to respond. After a jittered delay period with a blank screen, the next trial starts with the presentation of a trial‐start cue. (b) Illustration of the apparatus and the stimuli used in the experiment. The anode was attached to the middle phalanx of the index finger, the cathode on the proximal phalanx and the LED light on the distal phalanx.

The trial commenced with the display of a gray disk with a diameter of 40 pixels at the centre of the screen for a duration of 400 ms. Subsequently, a postcue period of varying duration between 750 and 1500 ms occurred. A 20‐ms stimulation period was subsequently initiated, during which the participant was tested in one of the four stimulus conditions with differences in the factors “light” (light–no light) or “stimulation” (stimulation–no stimulation). Therefore, the participants received a simultaneous 20‐ms LED and 0.3‐ms tactile stimulation together, light stimulation alone, tactile stimulation alone, or no stimulation. The tactile impulse was delivered 10 ms after the onset of the stimulation period and was therefore placed in the middle of the stimulation interval. A delay of 600 ms ensued, after which the response screen appeared, featuring a large question mark. The subjects were instructed to respond with their right hand by pressing one of four designated keys on the computer keyboard, corresponding to “definitely no” (index finger, [F] on the keyboard), “maybe no” (middle finger, [G]), “maybe yes” (ring finger, [H]), or “definitely yes” (little finger [J]). The keys for “definitely yes” and “definitely no” were labeled with green and red stickers, respectively. The order of the answer options was reversed (“definitely yes” on the left and “definitely no” on the right) depending on the response order, which was balanced across groups. A variable intertrial interval of 500–1000 ms preceded the next trial (see Figure 2a). The experiment comprised four blocks, each containing 100 trials, with each stimulus condition presented 25 times within each block. To prevent persistent floor or ceiling effects, the threshold procedure was repeated after a block if the hit rate in the touch‐only condition fell below 10% or exceeded 90%.

### 2.4 EEG recording and processing

EEG was recorded with 64 electrodes according to the 10–20 system using an active system (ActiveTwo, Biosemi). Three external electrodes were placed at the outer canthi of both eyes and below the left eye to detect eye movement (electrooculography; EOG). Two additional electrodes were placed at the left and right mastoids, which served as reference electrodes. For electrocardiography (ECG), three electrodes were placed on the left hip, right ankle, and right collarbone, forming an Einthoven's triangle. We performed EEG processing using the EEGLAB version 2019.1 (Delorme & Makeig, 2004) and Fieldtrip (Oostenveld et al., 2011) toolboxes running within the MATLAB R2018a environment. The signals were digitized at a sampling rate of 1024 Hz with 24‐bit A/D conversion. Data were resampled to 500 Hz, and continuous EEG signals were filtered via a high‐pass finite impulse response (FIR) filter with a low‐cut frequency at 0.5 Hz (filter‐order = 6144) and a low‐pass FIR filter at a high‐cut frequency at 30 Hz (filter‐order = 102). Flat electrodes without a signal for prolonged periods were removed (mean ± *SD*: 2 ± 2 electrodes), and electrodes were re‐referenced to the average reference. Major artifacts such as movement artifacts or voltage jumps were removed manually (continuous data points removed per individual: 3.82% ± 2.73%) before an independent component analysis (ICA) algorithm (infomax) was used to decompose the data into independent components. The components were classified via the EEGLAB plugin ICLabel version 1.2.5 and removed if the probability of being an eye component exceeded 85% while the probability of being a brain component fell below 5% (components removed: 2.91 ± 1.28). The data were then segmented into epochs from 1750 ms before until 1000 ms after potential stimulation onset, with subsequent baseline removal with a baseline between −200 and 0 ms before potential stimulation. Artifact rejection was automated using threshold rejection. Epochs exceeding ±100 μV were rejected, as were epochs exceeding a slope of 75 μV across a sliding time window of 1 s (epochs rejected: 3.59% ± 6.43%). Rejection rates were compared via *t*‐tests across stimulation conditions, response outcomes, and groups. No significant differences in rejection rates were found (all p‐values > .21).

The total time–frequency power was estimated in the range from 2 to 30 Hz in 1 Hz steps via a single Hann Taper with an adaptive window equal to four cycles per frequency calculated in 20 ms steps throughout the entire epoch. For later comparisons, the alpha frequency of 11 Hz was selected as in Craddock et al. (2017). Owing to the taper, the frequency of 11 Hz represents the alpha frequency band, including the neighboring frequency bands between 8.25 and 13.75 Hz, with a frequency smoothing of ±2.75 Hz and a window length of 364 ms. For time‐frequency baseline correction, time‐frequency data were subtracted and subsequently divided by a baseline lasting from −1625 to −1425 ms before stimulus onset (200 ms around average trial‐start cue onset at −1525 ms). The resulting values reflect the relative change in alpha power compared to the baseline period during the average cue onset time.

### 2.5 Behavioral signal detection analysis

For signal detection analysis, “maybe” and “definitely” responses were combined and grouped into “yes” and “no” responses. The hit rate, false alarm rate, and signal detection measures sensitivity d′ [z(hit) − z(false alarm)] and criterion c [−0.5 (z(hit) + z(false alarm))] were calculated based on the stimulus conditions (tactile stimulus present vs. absent) once for the light and no‐light condition. The sensitivity measure indicates the discrimination accuracy, i.e., how well a subject can correctly identify signals and their absence. The criterion represents how liberal or conservative the subject is to respond “yes” in general irrespective of the stimuli. A lower criterion indicates a lower threshold to say “yes” (Macmillan & Creelman, 2005). To correct for hit or false alarm rates of 0% and 100%, the formula 1/(2N) was used for 0% and 1–1/(2N) for 100%, with N being the number of trials (Macmillan & Creelman, 2005). The resulting signal detection measures were compared between groups with a 2 × 2 mixed ANOVA with “group” as a between‐subjects factor and “light” as a within‐subjects factor. Normality was assessed via the Shapiro–Wilk test. In cases of non‐parametric data, robust trimmed‐means methods from the R package WRS2 were used (Mair & Wilcox, 2020). The trimming level for robust tests was left at the default level of *γ* = .2. The significance level was set to *α* = .05, and effect sizes are reported as generalized eta square (*η*_g_^2^), Cohen's d (*d*), and Wilcox effect size (*r*). For the robust ANOVA, no effect size could be given. All the statistical analyses were performed via R 4.0.2 (R Core Team, 2020).

### 2.6 Somatosensory prestimulus alpha power and correlations

Baselined alpha values were compared between groups with the pre‐defined ROI based on Craddock et al. (2017) using values at a frequency of 11 Hz during the prestimulus time window between −600 and −200 ms before stimulus onset at the right centroparietal cluster contralateral to the stimulated hand (C2, C4, CP2, CP4) (Forschack et al., 2017). Comparisons between groups were made with a Mann–Whitney rank test because the distribution of baselined alpha values was non‐parametric. For exploratory purposes, absolute alpha values were also compared via the Mann–Whitney rank test.

To examine topographical neural differences in prestimulus alpha power over time, we explored cluster‐based differences in prestimulus alpha power between groups across all electrodes in the prestimulus time window from the end of the baseline period at −1425 ms before stimulus onset until stimulus onset at 0 ms. Using the Fieldtrip cluster‐based correction for multiple comparisons (Oostenveld et al., 2011), clusters were formed across time and space according to *t*‐values with a threshold of *p =* .05 and a minimum of two electrodes per cluster. For baselined alpha values, the sum of *t*‐values of a cluster was taken as a cluster‐level statistic. Owing to the strong non‐normal inter‐individual variability of absolute alpha power values, absolute alpha clusters were formed with Wilcox test statistics (threshold *p* = .05), and the sum of a cluster's Wilcox test statistics was taken as a cluster‐level statistic (Candia‐Rivera & Valenza, 2022). A null distribution of maximum‐sum clusters was formed with 2500 permutations. In each permutation, each data point was randomly allocated to a group, and the resulting maximum‐sum cluster was chosen. Clusters were considered significant if they fell below or above the 2.5th or 97.5th percentile of the reference permutation distribution.

Questionnaire, behavioral and alpha measures were added to an exploratory correlation analysis, in which variables were correlated with either Pearson statistics or Spearman statistics, depending on the normality of the variable determined by a Shapiro–Wilk test (*p* < .05). The correlations were conducted once across all participants (*n* = 64), once only within the meditation group (*n* = 31), and once within the non‐meditating group (*n* = 33). The results of this analysis have been added to the Supporting Information (A5: Correlation Tables).

### 2.7 Single‐trial analysis of somatosensory alpha power

To investigate trial‐by‐trial fluctuations in prestimulus alpha activity and associated behavioral measures, we related alpha measures to behavioral outcome measures with a generalized mixed‐effects model (GLMM). Following the methodology of Craddock et al. (2017), we analyzed how single‐trial alpha power in the right centroparietal cluster contralateral to the stimulated hand (C2, C4, CP2, CP4) at a frequency of 11 Hz within a time window from −600 to −200 ms before stimulus onset related to the response outcome. The relationship between single‐trial alpha power and response was modeled using a GLMM with a logistic function and the maximum random‐effects model, as recommended in Barr et al. (2013). The model was fitted via the *mixed* function from the afex package (Singmann et al., 2020) in R (R Core Team, 2020). The model was specified with the following syntax in R:

$$\begin{array}{c} \text{Response}\sim \text{stim}*\text{light}*\text{group}*\text{alpha}\\ \;\left(1+\text{stim}*\text{light}*\text{alpha}\;\|\;\text{subjectID}\right) \end{array}$$

The outcome variable response was collapsed; thus, “maybe yes” and “definitely yes” were coded as 1, and “maybe no” and “definitely no” were coded as 0. “Stim”, “light”, “group”, “alpha”, and their interactions were included as fixed effects, and the factors “stim”, “light”, and “alpha” were included as random slopes for the random effect of “subject”. The meditation group was coded as 1, and the control group was coded as 0. Correlations between random effects and intercepts were dropped, and contrasts were sum‐coded. For each participant, we normalized the estimates of alpha power to a mean of zero with a standard deviation of one. The contribution of individual terms to the model was measured by systematically removing each term from the model and comparing the goodness of fit with likelihood ratio tests. This provided a chi‐square statistic and a p‐value indicating the significant contribution of the term to the model. To fully replicate the trial‐by‐trial analysis conducted by Craddock et al. (2017), an analysis of the ipsilateral cluster (C1, C3, CP1, CP3) was also performed (see Supporting Information Table A4).

## 3 RESULTS

### 3.1 Questionnaire data

Contrary to our initial expectations, the group comparison yielded no statistically significant differences in the mean scores for trait mindfulness on the MAAS scale and no significant differences in physical symptoms on the PHQ‐15 scale. Despite the observed reduction in the “Difficulty Describing Feelings” scale among meditators, no notable discrepancy was identified in alexithymia (total TAS) between the two groups (see Table 1). The ERQ indicated lower expressive suppression in meditators but no significant difference in cognitive reappraisal (see Table 1). Meditators exhibited higher scores on almost all MAIA‐2 scales of interoceptive sensibility, even after Bonferroni correction for multiple comparisons: higher on “Noticing”, “Not‐Distracting”, “Not‐Worrying”, “Attention Regulation”, “Emotional Awareness”, “Self‐Regulation”, and “Body Listening”, but not “Trusting” after Bonferroni correction (see Table 1). Some scales showed slight deviations from normality; nevertheless, a Welch *t*‐test was used, as it is justified by the central limit theorem.

**TABLE 1:** Questionnaire data.

Measure	Experimental group (meditation)	Control group (reading)	Welch *t*‐test	Cohen's *d*
MAAS	4.28 (0.55)	4.16 (0.75)	*t*(59) = 0.74, *p* = .46	0.19
PHQ‐15	4.00 (3.57)^a^	3.21 (4.93)^a^	*t*(61) = 0.63, *p* = .52	0.10
ERQ Expressive Suppression	2.53 (0.82)	3.63 (1.49)	*t*(50) = 0.39, *p <* .001	0.94
ERQ Cognitive Reappraisal	4.78 (1.07)	5.17 (1.17)^b^	*t*(57) = −0.70, *p* = .49	0.18
TAS Difficulty Describing Feelings	9.81 (2.57)	11.76 (3.43)	*t*(59) = −2.58, *p* = .012, *p*_Corr_ = .037	0.64
TAS Difficulty Identifying Feelings	15.29 (3.63)	14.00 (9.00)^a^	*t*(60) = 0.71, *p* = .47	0.18
TAS Externally Oriented Thinking	15.68 (3.02)	16.91 (3.86)	*t*(60) = −1.42, *p* = .16	0.36
TAS Total Score	40.77 (6.48)	43.21 (9.24)	*t*(58) = −1.23, *p* = .22	0.31
MAIA‐2 Noticing	3.25 (0.38)^a^	2.68 (0.66)	*t*(54) = 3.89, *p <* .001, *p*_Corr_ < .01	0.97
MAIA‐2 Not‐Distracting	2.89 (0.60)	1.80 (0.82)	*t*(59) = 6.11, *p* < .001, *p*_Corr_ < .001	1.52
MAIA‐2 Not‐Worrying	2.60 (0.60)^b^	2.18 (0.56)	*t*(59) = 3.62, *p <* .001, *p*_Corr_ < .01	0.90
MAIA‐2 Attention Regulation	2.97 (0.44)	2.24 (0.54)	*t*(61) = 5.95, *p <* .001, *p*_Corr_ < .001	1.48
MAIA‐2 Emotional Awareness	3.32 (0.44)	2.73 (0.71)	*t*(54) = 3.97, *p <* .001, *p*_Corr_ < .01	0.97
MAIA‐2 Self‐Regulation	3.03 (0.38)	2.36 (0.74)	*t*(49) = 4.65, *p <* .001, *p*_Corr_ < .001	1.15
MAIA‐2 Body Listening	2.89 (0.42)	2.33 (0.66)^b^	*t*(45) = 4.64, *p <* .001, *p*_Corr_ < .001	1.15
MAIA‐2 Trusting	3.09 (0.62)	2.69 (0.82)	*t*(60) = 2.20, *p =* .032, *p*_Corr_ = .26	0.55

*Note*: Means (and SDs), statistics and effect sizes for the MAAS, PHQ‐15, ERQ, TAS, and all MAIA‐2 subscales reported for both groups. Bonferroni correction was used for the MAIA‐2 and TAS subscales. Medians and interquartile ranges are shown for non‐normally distributed data.

^a^ Positive skew.

^b^ Negative skew.

### 3.2 Behavioral data

Sensitivity measured by *d’* and criterion (*c*) were not normally distributed; thus, a robust mixed ANOVA using trimmed means was used (Mair & Wilcox, 2020). The robust ANOVA for sensitivity revealed a significant main effect for ‘light’, *Q*(1, 38) = 40.53, *p <* .001, and no main effect for ‘group’, *Q*(1, 29) = 0.01, *p =* .92, or an interaction between ‘light’ and ‘group’, *Q*(1, 38) = 0.11, *p =* .75. The criterion was found to be significantly lower when light was present, *F*(1, 62) = 43.80, *p* < .001, *η* ^*2*^ = .084, and a lower criterion in the meditator group was present, *F*(1, 62) = 4.19, *p* = .04, *η* ^*2*^ = .056. No interaction between ‘light’ and ‘group’ was found, *F*(1, 62) = 0.01, *p* = .93, *η* ^*2*^ < .001 (Table 2).

**TABLE 2:** Behavioral SSDT data.

	Hit rates (%)	False alarm rates (%)	Sensitivity (d′)	Criterion (c)
Experimental group (meditation)				
Light	70.26 (15.67)	6.00 (11.00)^a^	2.47 (1.30)^b^	0.47 (0.38)
No light	57.13 (17.36)	7.00 (11.25)^a^	1.98 (1.30)^b^	0.72 (0.42)
Control group (reading)				
Light	65.90 (18.62)	3.00 (5.00)^a^	2.34 (0.81)	0.67 (0.48)
No light	50.63 (19.70)	2.38 (4.00)^a^	1.96 (0.72)	0.93 (0.42)

*Note*: Means (and *SD*s) for hit rates, false alarm rates, sensitivity (d′) and criterion (c) reported for both groups. Medians and interquartile ranges are shown for non‐normally distributed data.

^a^ Positive skew.

^b^ Negative skew.

There was no significant difference in current strength for the 50% threshold values between the meditator and the non‐meditation group, with a mean current strength of 1.53 mA (*SD* = 0.49) for meditators and 1.41 (*SD* = 0.42) for non‐meditators, *t*(60) = 0.98, *p =* .33, *d* = 0.20, indicating that the results are not driven by differences in the current strength between groups.

### 3.3 Prestimulus alpha analysis

#### 3.3.1 Contralateral somatosensory ROI analysis

**FIGURE 3:**
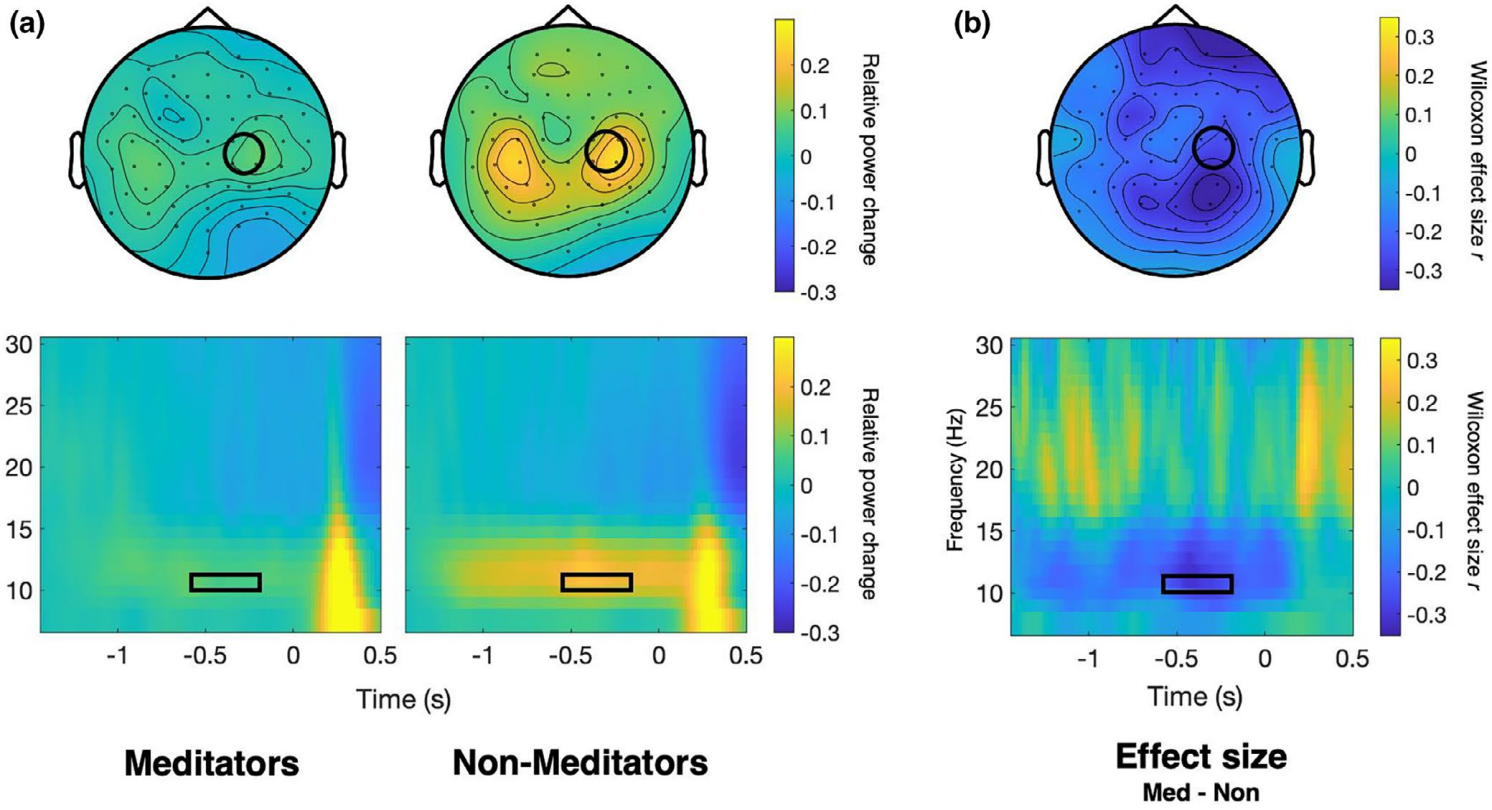
Baselined prestimulus alpha in meditators vs. controls. (a) Grand mean alpha power (11 Hz) for meditators and non‐meditators with baselined power values (relative change from baseline taken −1.625 to −1.425 s before stimulus onset) averaged across subjects. The top row shows the alpha power from −0.6 to −0.2 s before stimulus onset at 11 Hz, and the bottom row shows the time‐frequency power at the right centroparietal electrodes (C2, C4, CP2, CP4). The black circles and rectangles indicate a priori regions of interest. (b) The effect size, which indicates the difference in baselined alpha power between meditators and non‐meditators is expressed in Wilcoxon effect size *r*. Yellow areas indicate higher alpha activity in meditators, and blue areas indicate lower alpha activity in meditators. The difference between groups was significant for the ROI (*p* < .01).

A significant difference was observed at the pre‐defined somatotopic region representing the contralateral (stimulated) hand in baselined and absolute prestimulus alpha power between the two groups. Baselined prestimulus alpha power was significantly lower in meditators than in non‐meditators, with a median change of .02 in meditators and a median change of .14 in the non‐meditators' group, *U* = 708, *p* < .01, *r* = .33 (see Figure 3). We also tested the absolute prestimulus alpha power difference during the baseline and found a significant difference in absolute prestimulus alpha power during the baseline period (−1625 to −1425 ms), with a median alpha power of 0.81 μV^2^ in meditators and a median of 1.65 μV^2^ in the control group, *U* = 660, *p* = .046, *r* = .25. The absolute values of prestimulus alpha during the pre‐defined window of interest were significantly different between groups, with a median alpha power of 2.27 μV^2^ in non‐meditators and a median alpha power of 0.83 μV^2^ in meditators, *U* = 692, *p* = .015, *r* = .30. For a comparison of absolute vs. baselined time‐frequency data over time, see Supporting Information A2.

#### 3.3.2 Cluster‐based comparison of alpha power between groups

**FIGURE 4:**
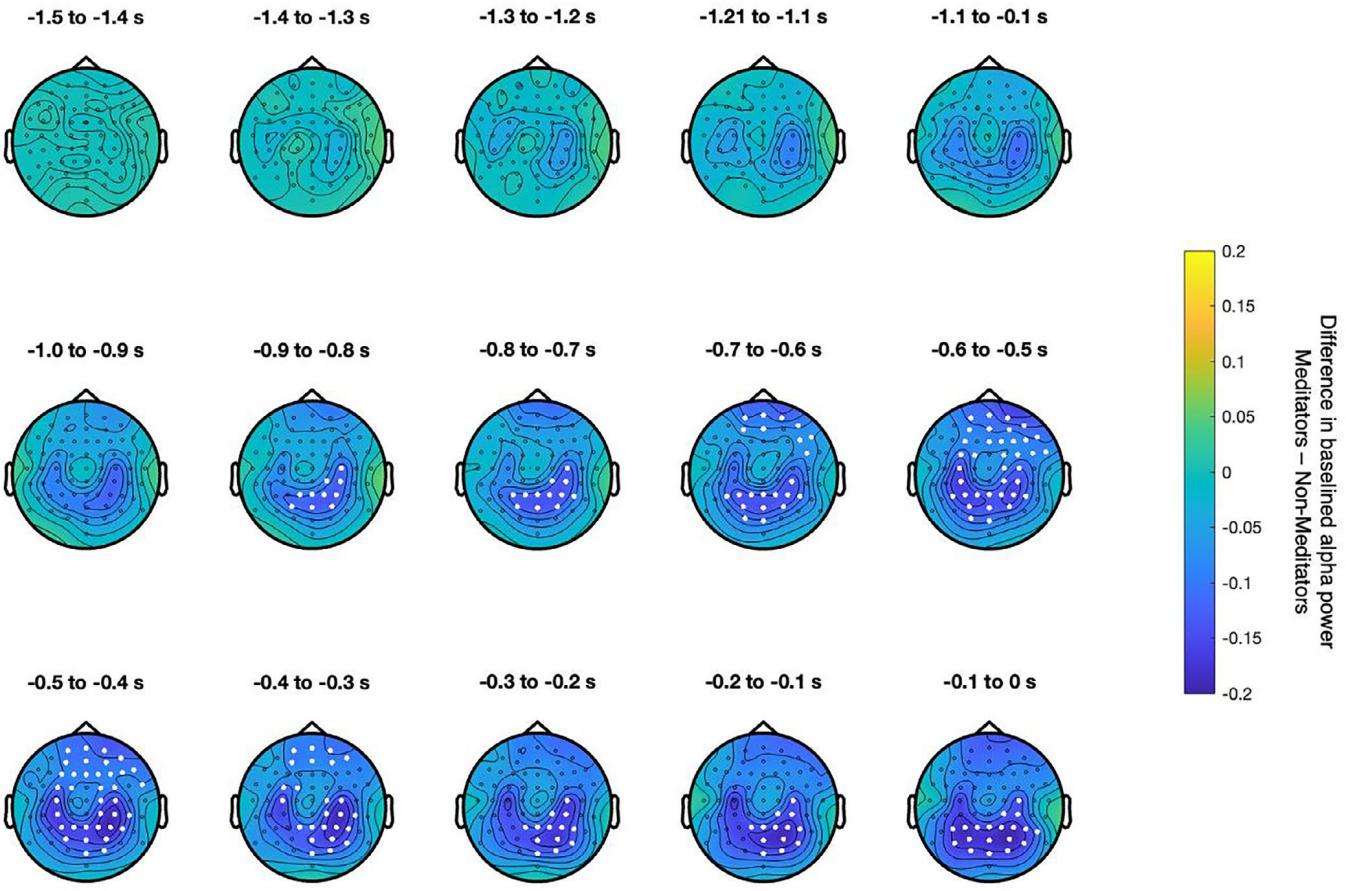
Cluster‐based differences in baselined prestimulus alpha power between groups. Baselined alpha power (relative change from baseline alpha power −1.625 to −1.425 s) at 11 Hz in meditators was subtracted from baselined alpha power from non‐meditators. Yellow indicates higher baselined alpha power in meditators, and blue indicates higher baselined alpha power in non‐meditators. Electrodes that were members of the significant cluster are highlighted in white. Non‐significant electrodes are marked with small black dots. Electrodes are highlighted when they were significant at any time throughout the period indicated above the respective topographical plot.

Using a cluster‐based comparison between groups over all electrodes with baselined prestimulus alpha power, a significant difference was revealed (*p* = .042). The cluster started at −900 ms in the mid‐right parietal region and remained until potential stimulation onset. A cluster in frontal regions appeared at −700 to −300 ms, connecting with the parietal cluster at −600 ms and disappearing at −300 ms before potential stimulus onset (Figure 4).

A cluster‐based comparison between groups with absolute prestimulus alpha power revealed a cluster that was marginally insignificant after 10,000 permutations (*p* = .057). The anatomical location of the cluster is mainly over the contralateral somatosensory cortex but also covers the ipsilateral somatosensory cortex from −900 ms before stimulus onset (see Supporting Information A3).

#### 3.3.3 Predicting behavioral performance in SSDT with prestimulus alpha

**FIGURE 5:**
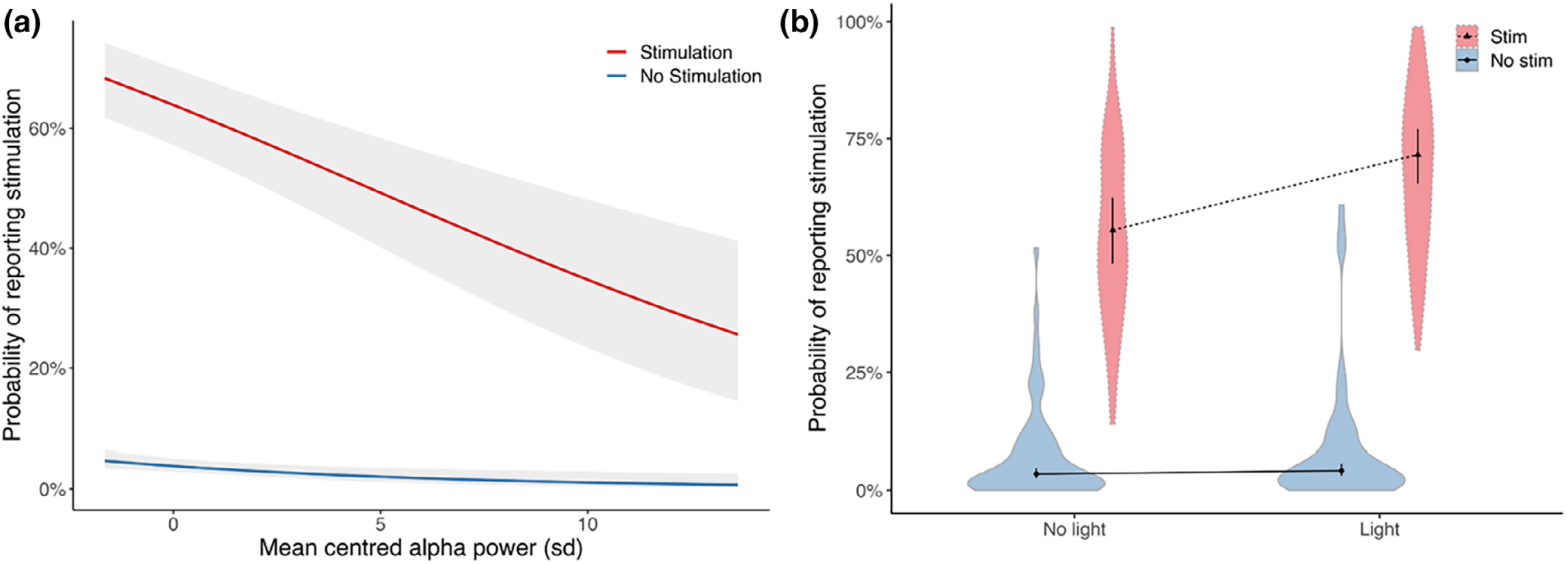
Relationship between prestimulus alpha power, stimulation, light, and reporting probability. (a) Relationship between prestimulus mean‐centered alpha power and the probability of reporting a stimulation. Lines indicate the estimated relationship between alpha power and the probability of reporting that a stimulation was present. The red line indicates the relationship during stim‐present trials (i.e., hit rate), and the blue line indicates the relationship during stim‐absent trials (i.e., false alarm rate). Shaded gray areas indicate 95% confidence intervals. The *x*‐axis depicts standardized individual *z*‐values of alpha activity. (b) Relationship between the factors ‘stimulation’ and ‘light’ and the probability of reporting a stimulation. Lines indicate the estimated relationship between the ‘stimulation’ and ‘light’ conditions and the probability of reporting a stimulation. The dotted line indicates that a stimulation was present, and the complete line indicates no tactile stimulation. Error bars represent 95% confidence intervals of estimates, and violin plots display the density of data points.

The trial‐by‐trial GLMM revealed that absolute prestimulus alpha power was a significant predictor of performance. In general, lower prestimulus alpha activity predicted a higher probability of reporting a tactile stimulation (see Figure 5a and Table 3). The main effects of ‘light’ and ‘stim’ and an interaction between ‘light’ and ‘stim’ were also found. A Bonferroni‐adjusted post‐hoc analysis demonstrated that the light condition increased the probability of reporting a stimulation only in the stimulation condition (*p* < .001) but not in the no‐stimulation condition (*p* > .11) (see Figure 5b). This finding is consistent with the behavioral findings, as ‘light’ increased the hit rate but not the false alarm rate (see Table 2). No interaction between ‘alpha’ and ‘stim’ was found in the model, indicating an effect of prestimulus alpha on the criterion and not sensitivity, and no interaction between ‘alpha’, ‘stim’, and ‘group’ was found, indicating no difference in the influence of ‘alpha’ on the response rate between groups. The trial‐by‐trial analysis of the ipsilateral side revealed similar results (see Supporting Information Table A4).

**TABLE 3:** Summary of fixed effects of GLMMs of contralateral prestimulus alpha power and its influence on reporting a stimulation.

	*Χ* ^*2*^	*p*	Odds ratio (95% CI)
Intercept			0.26 (0.22–0.32)
Stim	115.31	<.001	6.68 (5.41–8.25)
Light	29.70	<.001	1.25 (1.16–1.34)
Group	3.71	.054	1.22 (1.00–1.49)
Alpha	20.92	<.001	0.88 (0.84–0.93)
Stim:Light	18.65	<.001	1.14 (1.08–1.20)
Stim:Group	1.00	.32	0.90 (0.73–1.11)
Light:Group	0.57	.45	0.97 (0.91–1.04)
Stim:Alpha	0.03	.87	1.00 (0.95–1.06)
Light:Alpha	0.94	.33	0.97 (0.93–1.02)
Group:Alpha	0.10	.75	1.01 (0.96–1.06)
Stim:Light:Group	0.08	.78	1.01 (0.95–1.07)
Stim:Light:Alpha	0.02	.89	1.00 (0.95–1.05)
Stim:Group:Alpha	0.01	.91	1.05 (0.95–1.06)
Light:Group:Alpha	0.12	.73	1.00 (0.94–1.04)
Stim:Light:Group:Alpha	1.23	.27	1.03 (0.98–1.08)

*Note*: *Χ* ^2^ statistics and *p*‐values are derived from likelihood ratio tests. Odds ratios with 95% confidence intervals (CIs) are reported from the maximal model. Confidence intervals for the odds ratios were derived from coefficient standard errors.

## 4 DISCUSSION

In this cross‐sectional study, we examined the differences in somatosensory perception between regular meditation practitioners compared to non‐meditators by utilizing a somatosensory signal detection task (SSDT) in conjunction with simultaneous EEG recording to investigate prestimulus alpha. The meditation group exhibited a lower, more liberal criterion, indicating a greater propensity to report a stimulation sensation. However, there was no evidence of greater sensitivity. Furthermore, as anticipated, the baseline‐corrected prestimulus alpha power was found to be significantly lower in meditators over the somatosensory cortex. The same was observed with regard to absolute prestimulus alpha power. When a cluster‐based permutation test was employed, the baselined alpha power was significantly lower, and there was a trend towards reduced absolute alpha power. Both findings indicated a cluster encompassing the contralateral somatosensory cortex. The trial‐by‐trial analysis revealed a negative relationship between prestimulus alpha and the likelihood of reporting a stimulation sensation, showing that lower prestimulus alpha activity predicts a lowering of the criterion by increasing both, correct detections and false alarms (Craddock et al., 2017). At the questionnaire level, we found that meditators reported greater interoceptive sensibility (MAIA‐2) on all scales, lower difficulty describing feelings (TAS), and lower expressive suppression (ERQ). No significant differences between the groups were observed with regard to dispositional mindfulness (MAAS), overall alexithymia (TAS), or physical symptoms (PHQ‐15).

The behavioral group comparison revealed that meditators exhibited a lower criterion instead of increased sensitivity, indicating that they were more likely to report a stimulus regardless of its presence. When investigating the correlations between meditation practice and behavioral outcomes in the meditation group, a negative correlation between criterion during light and meditation session length and a negative correlation between criterion and meditation retreats yearly could be found, which is in line with the group difference, as meditation practice may be associated with a lower somatosensory decision criterion (see Supporting Information Table A5.2). A correlation between sensitivity (with and without light) and the number of meditation retreats was found, which could suggest a relationship between meditation practice during retreats and increased somatosensory accuracy. The total number of retreats may indicate thorough meditation expertise but could also be confounded with age. Overall, evidence points towards a relationship between meditation practice and a lowering of the criterion.

A lower criterion in meditators contrasts with previous research, where Mirams et al. (2013) reported an increase in sensitivity after a small meditation intervention relative to a story‐listening group. The difference in experimental procedure could be one factor accounting for the behavioral difference. Mirams et al. (2013)'s meditation group performed a body scan before the task, whereas our subjects performed the task without any previous meditation induction. Therefore, a state difference could account for the differences in sensitivity in Mirams et al. (2013), whereas in our study, trait differences in meditators could account for the difference in criterion. The meditators in our sample had a median meditation experience of more than 1500 h while the participants in the interventional design in Mirams et al. (2013) were novices who had only started learning how to meditate, with a total practice time of 1.75 hours. In a separate study, Mirams et al. (2012) reported a decrease in the criterion in the SSDT after participants performed a heartbeat perception task, indicating that interoceptive attention affects the somatosensory decision criterion (Mirams et al., 2012). As meditation practice involves a focus on body sensations and meditators scored higher on the Body Listening scale on the MAIA‐2, differences in interoceptive attention may have influenced the decision criterion in meditators. However, a lower criterion in the SSDT has also been related to higher somatisation and the associated false alarm rate has been positively correlated with physical symptom reports (Brown et al., 2010, 2012), possibly also describing maladaptive forms of somatosensory perception. As the light may cause ‘illusory’ perceptions, this has been discussed as evidence for somatosensory amplification, which could explain the prevalence of medically unexplained symptoms (Lloyd et al., 2008). Some evidence for this relationship can be found in the correlation analysis, as the criterion during the light condition was negatively correlated with the PHQ‐15 score when considering all participants; however, not when investigating groups individually, see Supporting Information Table A5.1. Similarly, in the control group, the criterion during light and sensitivity are positively correlated with MAIA‐2 Self‐Regulation suggesting that successful bodily self‐regulation is related to increased somatosensory detection accuracy and to a more conservative decision threshold during the light condition. The criterion was also found to be positively correlated with MAIA‐2 Not‐Distracting scale, indicating that the tendency to distract oneself from uncomfortable sensations is related to a more liberal criterion. However, these correlation results do not hold for the meditation group, suggesting different interpretations in the meditation population. The correlation analysis needs to be interpreted with caution because of its exploratory nature and the high number of tests. Nevertheless, this finding is in line with previous research regarding the SSDT, suggesting a relationship between the lower criterion and greater somatosensory attention and somatisation (Brown et al., 2010, 2012; Katzer et al., 2012).

Baseline‐corrected prestimulus alpha activity was found to be significantly lower in meditators within the ROI over the contralateral hand representation and when using the cluster‐based permutation test. The lower baselined prestimulus alpha activity over the somatosensory region in meditators was consistent with our hypothesis, and previous studies have shown that meditators exhibit enhanced attention modulation via alpha activity (Bailey et al., 2023; Kerr et al., 2011; Wang et al., 2020). Prestimulus alpha activity has been related to anticipatory gating of attention (Foxe & Snyder, 2011; Klimesch et al., 2007), and lower prestimulus alpha activity could reflect higher attentional “readiness”, as a state of lower alpha activity can generally be interpreted as a state of increased excitability. Prestimulus alpha decreases in response to a cue likely reflect the modulation of attention (Foxe & Snyder, 2011; Jensen & Mazaheri, 2010). In our paradigm, baselined alpha power represents the relative change in alpha power in response to the trial onset cue; therefore, one way to interpret lower baselined alpha power in meditators could be as greater attention modulation in the time domain. In the spatial domain, the baselined cluster difference was not specific to the somatosensory region itself (see Figure 4), possibly because of regional shifts in alpha power already during the baseline. The cluster in absolute alpha difference, however, seemingly reveals a cluster in our ROI, suggesting greater excitability in these task‐related regions (see Supporting Information A3). This finding is consistent with that of Kerr et al. (2011), who reported greater alpha modulation over the somatosensory cortex in meditators after an 8‐week MBSR intervention. In their review, Kerr et al. (2013) proposed that mindfulness meditation enhances top‐down modulation of alpha oscillations by increasing precision in the timing of thalamocortical inputs to the somatosensory cortex, and this mechanism is believed to be beneficial for the allocation of attention. As no control condition, such as “no attention,” was employed to specifically control for attention, the observed baselined alpha differences may not be solely attributable to attention. Notwithstanding, the lower baselined alpha activity is consistent with the predictions of the alpha modulation framework, which has been previously applied to research involving meditators (Bailey et al., 2023; Kerr et al., 2011; Wang et al., 2020).

Notably, in our study, the baseline itself was also significantly lower in meditators. These differences may reflect general differences in alpha power during the task or reactions from the previous trial. Although the effects of the preceding trial cannot be entirely ruled out, such an effect of the previous trial would have likely led to a greater recovery of alpha power during the prestimulus period, therefore, higher baselined alpha activity (see Supporting Information A2). Instead, meditators exhibited lower absolute alpha at the baseline level, as well as a lower baseline‐corrected prestimulus alpha. This indicates that alpha suppression was present both tonically and in response to the cue, in comparison to the non‐meditators. Most meditation‐related studies have reported an increase in alpha activity within meditative states compared with resting states (Brandmeyer & Delorme, 2018; Lomas et al., 2015), although decreases in alpha power have also been reported during the meditative state in meditators with long‐term practice (Amihai & Kozhevnikov, 2014; Cahn et al., 2010). In our study, we investigated upper alpha power at a frequency of 11 Hz using a task‐related design that required external attention to the task procedure. Part of the observed decrease in alpha power could also be attributed to a general state of alertness during the task as opposed to mind‐wandering. Several studies have shown that mind‐wandering is associated with increased global intertrial alpha power (Arnau et al., 2020; Compton et al., 2019; Jin et al., 2019) and that alpha power is negatively related to the self‐reported attentional state (Macdonald et al., 2011). This is consistent with the known benefits of meditation practice, such as reducing mind‐wandering and increasing presence (Kok & Singer, 2017; Malinowski, 2013; Mrazek et al., 2012; Verhaeghen, 2017). Additionally, the specific type of meditation, Vipassana meditation, practiced by a majority of the meditators in the sample involves focused and open attention (Hart & Goenka, 1987), which may also contribute to the observed effect, as lower alpha activity may reflect greater excitability and an open attentional state without mind‐wandering and decreased filtering.

Another way of interpreting our findings is that meditators show increased bottom‐up processing rather than top‐down processing, as mindful attention is described as seeing and perceiving things without extensive elaboration and prior expectations (Guendelman et al., 2017). In terms of predictive processing, this could be translated as a reduction in precision weighting on priors, as perception relies less on predictions and more on the input given in the present moment (Farb et al., 2015; Lutz et al., 2019; Pagnoni, 2019). Alpha oscillations have been postulated to carry top‐down predictions (Carhart‐Harris & Friston, 2019; Mayer et al., 2016), and decreased alpha activity in meditators could indicate lower top‐down predictions than bottom‐up processing. The observed increase in response rate could reflect higher bottom‐up sensitivity with a simultaneous lack of perceptual framing through top‐down predictions, causing the classification of endogenous activity or interoceptive signals as stimulation.

The correlation analysis further corroborates understanding by revealing a positive correlation between baselined alpha power and ERQ Expressive Suppression and TAS Difficulty Describing Feelings in the meditation group and across all participants, whereas a positive correlation between baselined alpha power and the total TAS score was also found across the whole sample (Supporting Information A5). Taken together, these findings suggest that baselined prestimulus alpha power may indicate emotional unawareness and emotional suppression. Translated into the alpha modulation framework, decreased alpha modulation may hinder the ability to tune attention to emotions and their bodily manifestations, potentially impairing the accurate recognition and processing of emotional experiences (Kerr et al., 2013). The implied negative associations between baselined alpha power and MAIA‐2 scales of interoceptive sensibility were not significant, suggesting a closer relationship with emotional processing than with interoceptive processing directly. The evidence regarding absolute alpha and questionnaire measures is less clear. A negative correlation between absolute contralateral prestimulus alpha power and MAIA‐2 Self‐Regulation was significant across all participants, which is in line with the hypothesis that bodily emotion regulation is increased with lower alpha activity. A positive correlation between MAIA‐2 Body Listening and absolute alpha power in the meditation sample, however, is contrary to the previous finding. Since both findings are not confirmed by other related findings, the possibility is likely that these are statistical artifacts, as the chance for Type‐1 error is increased owing to the number of tests.

The trial‐by‐trial analysis provided further evidence regarding the interpretability of absolute alpha power, which revealed that the likelihood of reporting a touch sensation increased as alpha power decreased, both over the ipsilateral and contralateral somatosensory cortices. This finding is consistent with the results of Craddock et al. (2017), who observed a correlation between higher response rates and lower alpha power in the ipsilateral and contralateral somatosensory cortices and, additionally, established causality between alpha oscillations and criterion with transcranial alternating current stimulation (tACS) (Craddock et al., 2019). No interaction was found between the stimulation condition and alpha power, which would indicate an increase in sensitivity with lower alpha power. This interaction was present neither for meditators nor for non‐meditators, showing that the same mechanism of cortical excitability onto the decision criterion applies to meditators and controls. Therefore, lower alpha activity in meditators during the prestimulus time window could explain the behavioral difference in the criterion. The trial‐by‐trial analysis also revealed a trend that being a meditator alone influenced the propensity to report a touch sensation (*p* = .054). This aligns with the signal detection data, as it revealed a group difference in the criterion between groups. The alpha power was normalized for each participant; therefore, the factor ‘alpha’ exclusively accounted for changes in response rate based on trial‐by‐trial fluctuations in prestimulus alpha power, regardless of individual differences. When individual average alpha power was correlated with the criterion, however, no significant relationship was observed (see Supporting Information A5). Overall, the trial‐by‐trial analysis confirmed the assumption that prestimulus alpha power has a filtering function and is inversely related to cortical excitability (Jensen & Mazaheri, 2010; Klimesch et al., 2007). Similarly, as discussed above, decreased alpha may indicate increased anticipatory attention or alertness, which increases the chance of detecting a stimulus but also increases the chance of falsely reporting the detection of a stimulus. During lower alpha, subjects may be more susceptible to feeling small endogenous sensations stemming from the finger, increasing the chance of both detecting stimulation and misinterpreting body sensations as stimulations.

On the questionnaire level, overall higher scores on the MAIA‐2 scales for interoceptive sensibility indicate that meditators show overall higher subjective body awareness. This is in line with previous research in which meditators reported greater interoceptive sensibility (Bornemann et al., 2014; Heeter et al., 2017; Mehling et al., 2012). When investigating only the meditation group and correlations between MAIA‐2 scores and measures of practice intensity, the exploratory correlation analysis revealed mixed results. While MAIA‐2 Noticing significantly increased with increasing meditation session length, Not‐Worrying decreased with increasing meditation session length (see Supporting Information Table A5.2). During longer meditation sessions, increased pain through sitting could be related to increased noticing of body sensations but also increased worrying about them. Alternatively, increased worries could also cause practitioners to meditate for longer to calm down their mind. Since the findings are associative, causality is unclear. The overall score of Not‐Worrying in meditators was higher than that in controls; therefore, the positive effects of meditation outweigh the negative effects. Dispositional mindfulness, as measured by the MAAS, was not found to be increased in meditators, which is unexpected given the known link between meditation practice and increased trait mindfulness after an intervention (Brown et al., 2011; Chambers et al., 2008; Krygier et al., 2013). One possible explanation is that the MAAS assesses reports of attentional lapses and that this measure may not be suitable for long‐term meditation practitioners (Grossman, 2011). We did not find a significant difference in physical symptom reports as measured by the PHQ‐15, although we expected that meditation practice would have beneficial effects on relieving physical symptoms (Lakhan & Schofield, 2013). This may have occurred due to higher awareness of bodily sensations in meditators in general, as it has also been reported with higher scores on the MAIA‐2 Noticing scale. Increased body awareness may include both pleasant and unpleasant sensations and may result in similar reports of physical symptoms in meditators compared to non‐meditators. Investigating this effect further with correlations within the meditation group, physical symptoms were positively correlated with meditation years, meditation session length, and the compound measure of total meditation hours (Supporting Information Table A5.2). This is unexpected, as more meditation practice was expected to decrease physical symptoms more (Lakhan & Schofield, 2013). However, unwanted effects of meditation do not seem uncommon. Cebolla et al. (2017) reported that increased meditation session length is related to increased self‐reports of unwanted effects and that 25% of practitioners reported some (transitory) unwanted effects. The overall TAS score did not differ between the groups, but the meditation group overall reported lower Difficulty Describing Feelings. Within the meditation group itself, the overall TAS score was positively correlated with meditation session length, meditation sessions weekly, the resulting value of meditation hours per week, and total meditation hours. A similar pattern can be found for the Expressive Suppression score on the ERQ, which indicates the use of a maladaptive emotion regulation strategy that suppresses negative states. Overall, the meditation group reported lower Expressive Suppression, however, within the meditation group, it was positively correlated with the number of meditation sessions weekly. Generally, positive effects of meditation, if present, may, therefore, decrease with increased intensity of practice. Although adverse effects have been increasingly discussed in recent studies (Farias et al., 2020; Lambert et al., 2023), the mechanisms are not fully understood, as some negative side effects may also be part of the process (Lindahl et al., 2020). Britton (2019) suggested that positive benefits may follow an inverted U‐shape where positive phenomena can be reduced with increased intensity of practice. Possibly, our inclusion criterion of 5 hours of practice per week may be close to optimal, as more practice correlated with decreased benefits in the meditation group. Further research into the unwanted effects of meditation practice may be needed to understand the causes of these relationships. Intense meditation practice could cause unwanted effects, or individuals with increased physical symptoms could practice more because of its widely acknowledged positive impact on mental health. Overall, higher scores on interoceptive sensibility scales, lower scores on emotional suppression, and difficulty describing feelings, together with a positive correlation between meditation practice and noticing body sensations, highlight the positive effects of meditation practice on body awareness and emotional awareness. While correlations between practice time and physical symptoms, alexithymia, and worrying about body sensations underscore the complexity of its impacts, these findings also open interesting avenues for further research.

Several factors should be taken into consideration when our results are interpreted. The development of a comprehensive model that accurately explains the correlation between mindfulness, body awareness, and interoception is hindered by the limitations of our experimental design: we used a quasi‐experimental cross‐sectional design, with a control group of readers, which may have influenced the differences observed between the groups by factors specific to the control group. For example, readers may have exhibited more mind‐wandering due to the nature of reading novels, which requires visualizing images in addition to perceiving the present world. As we did not follow an interventional design, we cannot conclude whether the group differences are due to the body‐based meditation practice itself or if the effects are driven by other lifestyle factors in meditation practitioners. A longitudinal design could eliminate these factors and ensure that the differences observed are specifically related to meditation practice. Moreover, compared to Mirams et al. (2013), our false alarm rate was generally low and not influenced by the light. The type of touch stimulation used in our paradigm could account for those differences. We used electrical stimulation, whereas Mirams et al. (2013) used vibrotactile stimulation. Studies using vibrotactile stimulation have shown higher false alarm rates than those using electrical stimulation, and this false alarm rate usually significantly increased with the light in vibrotactile paradigms (Brown et al., 2010, 2012; Johnson et al., 2006; Lloyd et al., 2008).

With respect to the clinical implications of these findings, we motivate future studies to further investigate the influences of meditation practice and its effects on somatosensory processing and physical symptoms in terms of predictive processing. For example, stimulus expectations could be integrated to test the effects of prior expectations on somatosensory processing between meditators and controls. Aberrant priors have been proposed as the cause of medically unexplained symptoms (Pezzulo et al., 2019; van den Bergh et al., 2017), and further investigations to decrease their influence by training attentional control towards body sensations could be of interest; however, it is also essential to consider the adverse effects of meditation practice. This would warrant specific modifications to the type of meditation practices and their dosage according to specific populations (Goldberg, Lam, et al., 2022).

## 5 CONCLUSION

Overall, our findings revealed lower prestimulus alpha activity over the somatosensory cortex in meditators relative to controls, suggesting greater somatosensory attention modulation in long‐term meditation practitioners than in controls. Furthermore, we found a lower decision threshold to report electro‐tactile sensations, indicated by a decrease in the signal detection criterion but not an increase in somatosensory detection accuracy. This finding is in line with the trial‐by‐trial analysis, which related trial‐by‐trial fluctuations in alpha activity to somatosensory decision‐making. Lower prestimulus alpha activity was predictive of a greater propensity to respond “yes”; therefore, lower prestimulus alpha implies a lower decision threshold. Part of the behavioral difference in response between groups could, therefore, be explained via the difference in prestimulus alpha activity. Reports of greater interoceptive sensibility were found and less suppressive emotion regulation was reported; taken together, the results suggest that although meditators report greater body awareness, somatosensory signal detection is altered in more complex ways than merely an increase in accuracy. This study is the first to document differences in somatosensory signal detection processing and suggest greater prestimulus alpha modulation in a cross‐sectional design including long‐term meditators.

## AUTHOR CONTRIBUTIONS

**Maik Mylius:** Conceptualization; data curation; formal analysis; investigation; methodology; software; visualization; writing – original draft; writing – review and editing. **Simon Guendelman:** Conceptualization; project administration; supervision; writing – review and editing. **Fivos Iliopoulos:** Conceptualization; methodology; resources; supervision; writing – review and editing. **Vittorio Gallese:** Funding acquisition; supervision. **Laura Kaltwasser:** Conceptualization; funding acquisition; methodology; project administration; supervision; writing – review and editing.

## FUNDING INFORMATION

This work was supported by the Einstein Foundation (Einstein Center for Neurosciences Berlin).

## CONFLICT OF INTEREST STATEMENT

We have no known conflict of interest to disclose.

## Supporting information


Data S1.
**FIGURE A2.** Median alpha power between groups over the contralateral somatosensory cortex over time. Lines indicate the median alpha power between groups over the somatosensory cortex (C2, C4, CP2, CP4). The shaded grey areas indicate the a‐priori time period of interest. (a) Lines indicate the median absolute alpha power over time per group. (b) Lines indicate median baselined alpha power between groups. The baseline was taken at the average time of trial cue‐onset from −1.625 to −1.425 ms before stimulus onset, and baselined values represent the relative change from baseline.
**FIGURE A3.** Cluster‐based differences in absolute prestimulus alpha power between groups. The reported effect size, expressed in Wilcoxon effect size *r*, indicates the differences in absolute alpha power at 11 Hz between meditators and readers. Blue indicates lower alpha activity in meditators. Electrodes that were members of the marginally insignificant cluster (*p* = .057) are highlighted in white. Non‐significant electrodes are marked with small black dots. Electrodes are highlighted when they were part of the cluster at any time throughout the period indicated above the respective topographical plot.
**TABLE A4.** Summary of fixed effects of GLMMs of ipsilateral prestimulus alpha power and its influence on reporting a stimulation.
**TABLE A5.1.** Correlations across all participants (*n* = 64).
**TABLE A5.2.** Within‐group correlations across the meditation group (*n* = 31).
**TABLE A5.3.** Within‐group correlations across the non‐meditator group (*n* = 33).

## Data Availability

The data that support the findings of this study are available upon request from the corresponding author. The data are not publicly available due to privacy or ethical restrictions.
