# Supplementary material for: Meditation expertise influences response bias and prestimulus alpha activity in the somatosensory signal detection task
Source: Psychophysiology. 2024 Nov 18;62(2):e14712. doi: 10.1111/psyp.14712 (PMC11870818; doi:10.1111/psyp.14712)
Supplement: Supplementary file 1 — Data S1. FIGURE A2. Median alpha power between groups over the contralateral somatosensory cortex over time. Lines indicate the median alpha power between groups over the somatosensory cortex (C2, C4, CP2, CP4). The shaded grey areas indicate the a‐priori time period of interest. (a) Lines indicate the median absolute alpha power over time per group. (b) Lines indicate median baselined alpha power between groups. The baseline was taken at the average time of trial cue‐onset from −1.625 to −1.425 ms before stimulus onset, and baselined values represent the relative change from baseline. FIGURE A3. Cluster‐based differences in absolute prestimulus alpha power between groups. The reported effect size, expressed in Wilcoxon effect size r, indicates the differences in absolute alpha power at 11 Hz between meditators and readers. Blue indicates lower alpha activity in meditators. Electrodes that were members of the marginally insignificant cluster (p = .057) are highlighted in white. Non‐significant electrodes are marked with small black dots. Electrodes are highlighted when they were part of the cluster at any time throughout the period indicated above the respective topographical plot. TABLE A4. Summary of fixed effects of GLMMs of ipsilateral prestimulus alpha power and its influence on reporting a stimulation. TABLE A5.1. Correlations across all participants (n = 64). TABLE A5.2. Within‐group correlations across the meditation group (n = 31). TABLE A5.3. Within‐group correlations across the non‐meditator group (n = 33). [file PSYP-62-e14712-s001.docx]

**A1: Thresholding procedure**

First, a rough estimate of the perceptual threshold was assessed by manually sending electrical pulses of varying strength repeatedly and asking the participant verbally, whether a sensation was perceived or not. The participant responded verbally, with “Yes” or “No”. The experimenter started with low intensity and, by receiving verbal feedback, assessed a rough estimate of the decision boundary. This was used as a starting point for an automated staircase procedure.

During the automated adaptive staircase procedure, a series of stimuli of varying intensity were presented to the participants, who were instructed to visually attend to their left hand and to indicate whether they perceived a sensation. The beginning of a trial was marked by a grey disk at the centre of the screen with a diameter of 40 pixels shown for 500 ms. Afterwards, a black screen was shown for a randomly varying post-cue period from 1000 ms to 1300 ms, and a stimulation with a length of 0.3 ms was given, followed by a waiting period of 499.7 ms. A response screen displayed the letters “Y ? N”, and the participants were able to respond either with “yes” or “no” using their right index finger or their ring finger on a computer keyboard. The response condition was balanced across participants. To estimate the decision threshold, a 1-up/1-down adaptive staircase procedure was implemented to select intensity levels for the stimulations (Levitt, 1971). One descending staircase started 0.5 milliamperes (mA) higher than the estimated threshold, whereas the other ascending staircase started 0.5 mA lower than the threshold. The intensity was adjusted in 0.05 mA steps based on the response. To prevent habituation, 0-mA trials were induced, which occurred between staircase trials with a probability of 30%. The stopping criterion for the staircases was eight reversals, and the decision threshold was computed by averaging the intensities of the last five reversals.

To test the validity of the resulting threshold, 13 trials were generated, of which 10 test trials had the estimated threshold intensity and three zero-trials presented no current. If the participant responded with four to six hits for the 10 test trials, the threshold was granted as valid and used for the subsequent SSDT. If the test failed, the staircase procedure was run again, and the new resulting threshold was computed as the average threshold between all staircase runs. If the third threshold failed to pass the validation test, the script stopped, and a manual staircase script was run where the experimenter specified an estimated threshold until it passed the validation test (Johnson et al., 2006).

**A2: Absolute vs. baselined alpha frequency**


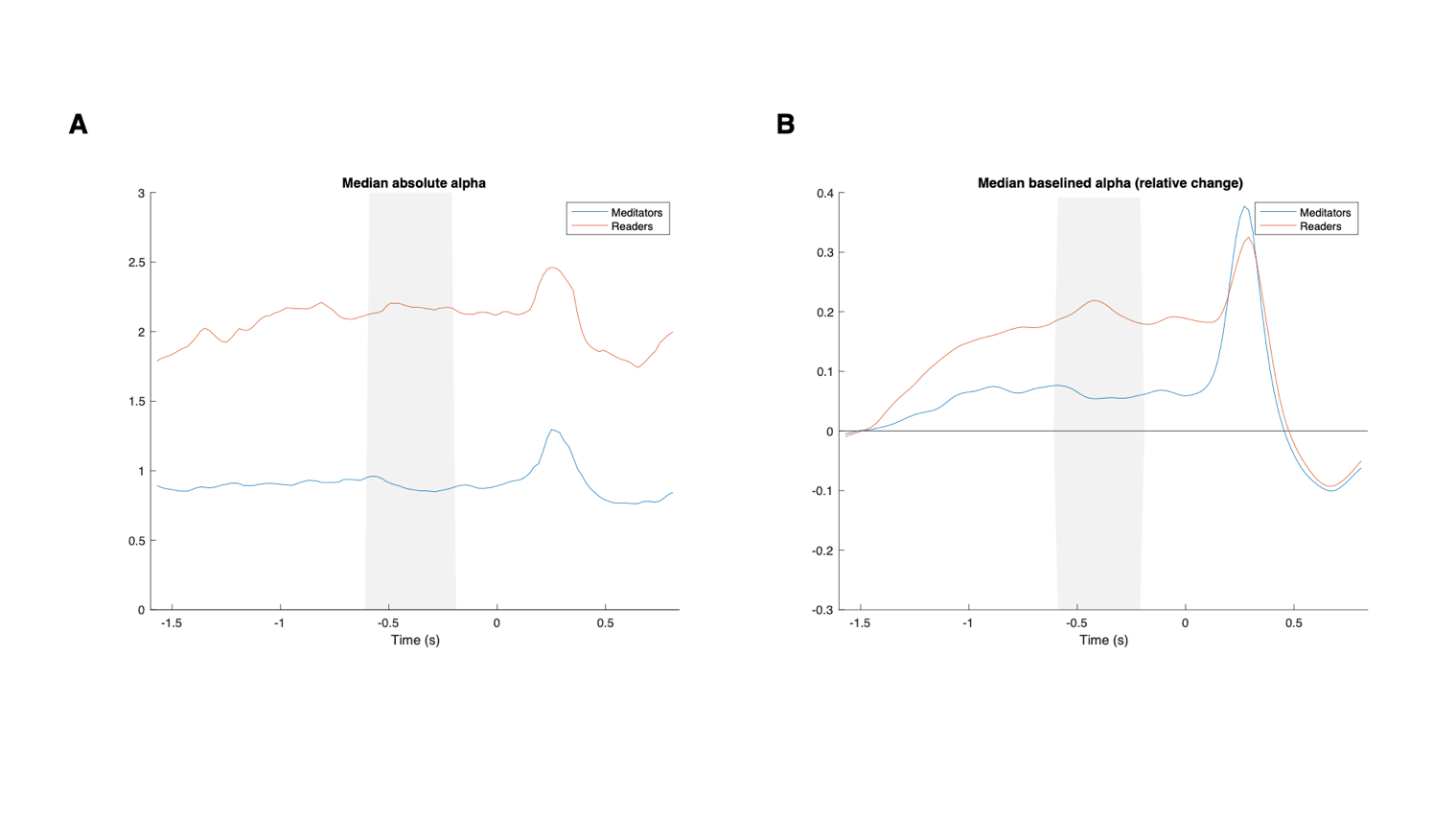


**Figure A2: Median alpha power between groups over the contralateral somatosensory cortex over time.** Lines indicate the median alpha power between groups over the somatosensory cortex (C2, C4, CP2, CP4). The shaded grey areas indicate the a-priori time period of interest. A) Lines indicate the median absolute alpha power over time per group. B) Lines indicate median baselined alpha power between groups. The baseline was taken at the average time of trial cue-onset from -1.625 ms to -1.425 ms before stimulus onset, and baselined values represent the relative change from baseline.

**A3: Cluster-based differences in absolute alpha power between groups**

**
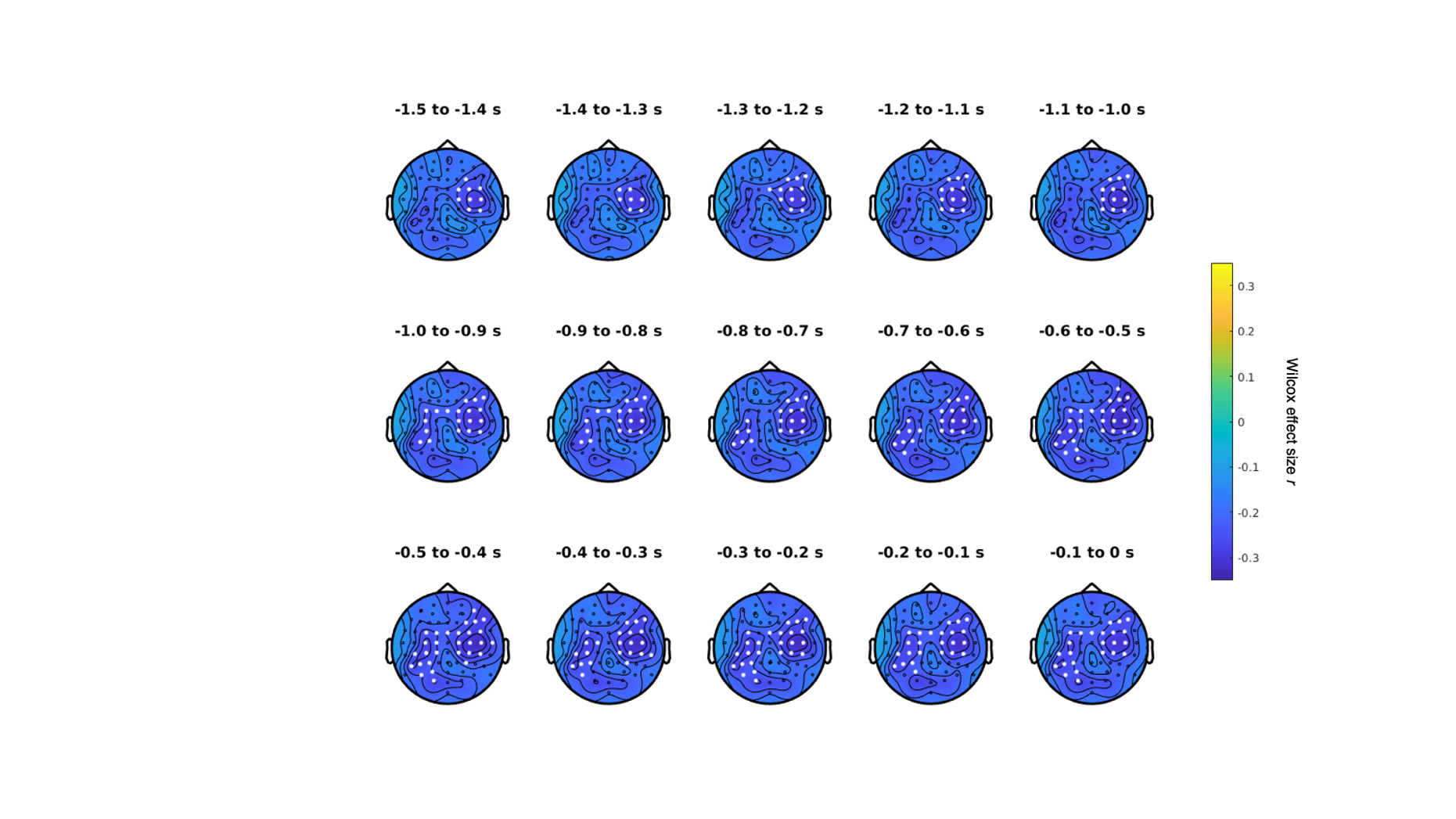
**

**Figure A3: Cluster-based differences in absolute prestimulus alpha power between groups.** The reported effect size, expressed in Wilcoxon effect size r, indicates the differences in absolute alpha power at 11 Hz between meditators and readers. Blue indicates lower alpha activity in meditators. Electrodes that were members of the marginally insignificant cluster (*p* = .057) are highlighted in white. Non-significant electrodes are marked with small black dots. Electrodes are highlighted when they were part of the cluster at any time throughout the period indicated above the respective topographical plot.

**A4: Ipsilateral trial-by-trial analysis**

**Table A4
Summary of fixed effects of GLMMs of ipsilateral prestimulus alpha power and its influence on reporting a stimulation.** Χ^2^ statistics and p-values are derived from likelihood ratio tests. Odds ratios with 95% confidence intervals (CIs) are reported from the maximal model. Confidence intervals for the odds ratios were derived from coefficient standard errors.

|  | *Χ^2^* | *p* | Odds ratio (95% CI) |
| --- | --- | --- | --- |
| Intercept |  |  | 0.26 (0.22 – 0.32) |
| Stim | 115.21 | < .001 | 6.67 (5.40 – 8.24) |
| Light | 29.42 | < .001 | 1.25 (1.16 – 1.34) |
| Group | 3.70 | .055 | 1.22 (1.00 – 1.49) |
| Alpha | 20.86 | < .001 | 0.89 (0.85 – 0.94) |
| Stim:Light | 18.39 | < .001 | 1.14 (1.08 – 1.20) |
| Stim:Group | 1.00 | .32 | 0.90 (0.73 – 1.11) |
| Light:Group | 0.44 | .51 | 0.99 (0.91 – 1.05) |
| Stim:Alpha | 1.81 | .18 | 1.03 (0.98 – 1.09) |
| Light:Alpha | 0.21 | .64 | 0.99 (0.94 – 1.04) |
| Group:Alpha | 0.03 | .87 | 1.00 (0.95 – 1.05) |
| Stim:Light:Group | 0.03 | .86 | 1.00 (0.95 – 1.06) |
| Stim:Light:Alpha | 0.01 | .93 | 1.00 (0.95 – 1.05) |
| Stim:Group:Alpha | 0.31 | .58 | 1.01 (0.96 – 1.07) |
| Light:Group:Alpha | 1.13 | .29 | 1.03 (0.98 – 1.08) |
| Stim:Light:Group:Alpha | 0.02 | .88 | 1.00 (0.95 – 1.05) |

**A5: Correlation Tables**

**Table 5.1
Correlations across all participants (n = 64).** Means, standard deviations and Pearson correlation coefficients are shown. The variables in blue are non-normally distributed and were correlated with Spearman correlations. Medians and interquartile ranges are shown for non-normally distributed data (marked with ^a^ for positive skew and ^b^ for negative skew).

| Variable | *M* | *SD* | 1 | 2 | 3 | 4 | 5 | 6 | 7 | 8 | 9 | 10 | 11 | 12 | 13 | 14 | 15 | 16 | 17 | 18 | 19 | 20 |
| --- | --- | --- | --- | --- | --- | --- | --- | --- | --- | --- | --- | --- | --- | --- | --- | --- | --- | --- | --- | --- | --- | --- |
| 1. MAAS | 4.22 | 0.66 |  |  |  |  |  |  |  |  |  |  |  |  |  |  |  |  |  |  |  |  |
| 2. TAS Difficulty Describing Feelings | 10.0^a^ | 4.0^a^ | -.43 ** |  |  |  |  |  |  |  |  |  |  |  |  |  |  |  |  |  |  |  |
| 3. TAS Difficulty Identifying Feelings | 14.91 | 4.19 | -.51 ** | .41** |  |  |  |  |  |  |  |  |  |  |  |  |  |  |  |  |  |  |
| 4. TAS Total Score | 42.03 | 8.05 | -.42 ** | .67** | .78** |  |  |  |  |  |  |  |  |  |  |  |  |  |  |  |  |  |
| 5. MAIA-2 Noticing | 3.0^b^ | 0.5^b^ | .25* | -.36 ** | -.09 | -.13 |  |  |  |  |  |  |  |  |  |  |  |  |  |  |  |  |
| 6. MAIA-2 Not-Distracting | 2.33 | 0.90 | .40** | -.36 ** | -.15 | -.16 | .38** |  |  |  |  |  |  |  |  |  |  |  |  |  |  |  |
| 7. MAIA-2 Not-Worrying | 2.4^b^ | 0.8^b^ | .32** | -.35 ** | -.22 | -.39 ** | .11 | .38** |  |  |  |  |  |  |  |  |  |  |  |  |  |  |
| 8. MAIA-2 Attention Regulation | 2.60 | 0.61 | .29* | -.38 ** | -.11 | -.23 | .64** | .49** | .44** |  |  |  |  |  |  |  |  |  |  |  |  |  |
| 9. MAIA-2 Emotional Awareness | 3.0^b^ | 0.65^b^ | .08 | -.29* | -.06 | -.15 | .56** | .41** | .37** | .67** |  |  |  |  |  |  |  |  |  |  |  |  |
| 10. MAIA-2 Self-Regulation | 2.75^b^ | 0.75^b^ | .11 | -.28* | .03 | -.18 | .41** | .37** | .34** | .59** | .61** |  |  |  |  |  |  |  |  |  |  |  |
| 11. MAIA-2 Body Listening | 2.67^b^ | 0.67^b^ | .04 | -.16 | .08 | -.08 | .47 ** | .40** | .26* | .61** | .62** | .57** |  |  |  |  |  |  |  |  |  |  |
| 12. MAIA-2 Trusting | 3.0^b^ | 1.0^b^ | .09 | -.19 | -.24 | -.22 | .22 | .32* | .25* | .32** | .43** | .28* | .44** |  |  |  |  |  |  |  |  |  |
| 13. ERQ Cognitive Reappraisal | 4.87 | 0.95 | .02 | -.12 | -.20 | -.30* | -.15 | -.16 | .13 | -.09 | .12 | .11 | .07 | .27* |  |  |  |  |  |  |  |  |
| 14. ERQ Expressive Suppression | 3.0^a^ | 1.62^a^ | -.40 ** | .48** | .18 | .42** | -.11 | -.42 ** | -.36 ** | -.28* | -.18 | -.26* | -.13 | -.09 | -.08 |  |  |  |  |  |  |  |
| 15. PHQ-15 | 3.61^a^ | 4.0^a^ | -.41 ** | .22 | .30* | .26* | -.07 | -.24 | -.33 ** | -.17 | -.05 | .04 | -.01 | -.27* | -.06 | .16 |  |  |  |  |  |  |
| 16. Criterion | 0.87^b^ | 0.71^b^ | -.15 | .01 | -.18 | -.15 | -.16 | -.07 | -.02 | -.21 | -.04 | -.08 | -.20 | .23 | .06 | .19 | -.21 |  |  |  |  |  |
| 17. Criterion during Light | 0.57 | 0.45 | .07 | -.09 | -.06 | -.05 | -.19 | -.05 | .05 | .01 | -.11 | -.04 | -.20 | .14 | .00 | .08 | -.26* | .80** |  |  |  |  |
| 18. Sensitivity | 1.89 | 0.84 | -.14 | .01 | .08 | .02 | .02 | -.11 | .10 | .03 | .12 | .12 | .03 | -.00 | .11 | .02 | .02 | .22 | .23 |  |  |  |
| 19. Sensitivity during Light | 2.33^b^ | 1.02^b^ | -.08 | .07 | .03 | -.04 | .01 | -.10 | .00 | .01 | .08 | .07 | .01 | .03 | .09 | .07 | .02 | .09 | .16 | .86** |  |  |
| 20. Alpha Power Contralateral | 1.66^a^ | 3.35^a^ | -.02 | .12 | .08 | .07 | -.08 | -.09 | .18 | -.08 | -.13 | -.28* | -.02 | .09 | .03 | .18 | -.24 | -.06 | .05 | -.03 | -.06 |  |
| 21. Alpha Power Contralateral Baselined | 0.07^a^ | 0.29^a^ | -.15 | .34** | .13 | .28* | -.24 | -.16 | -.11 | -.12 | -.04 | -.18 | .01 | -.12 | -.11 | .40** | -.02 | -.08 | -.08 | .02 | .04 | .38** |

*M* and *SD* are used to represent the mean and standard deviation, respectively. Medians and interquartile ranges are shown for non-normally distributed data (marked with ^a^ for positive skew and ^b^ for negative skew). * indicates *p* < .05. ** indicates *p* < .01.

**Table A5.2**

**Within-group correlations across the meditation group (*n* = 31).** Means, standard deviations and Pearson correlation coefficients are shown. The variables in blue are non-normally distributed and were correlated with Spearman correlations. Medians and interquartile ranges are shown for non-normally distributed data (marked with a for positive skew and b for negative skew).

| Variable | *M* | *SD* | 1 | 2 | 3 | 4 | 5 | 6 | 7 | 8 | 9 | 10 | 11 | 12 | 13 | 14 | 15 | 16 | 17 | 18 | 19 | 20 | 21 | 22 | 27 | 28 | 29 | 30 | 31 |
| --- | --- | --- | --- | --- | --- | --- | --- | --- | --- | --- | --- | --- | --- | --- | --- | --- | --- | --- | --- | --- | --- | --- | --- | --- | --- | --- | --- | --- | --- |
| 1. Meditation Years | 4.5^a^ | 3.0^a^ |  |  |  |  |  |  |  |  |  |  |  |  |  |  |  |  |  |  |  |  |  |  |  |  |  |  |  |
| 2. Meditation Session Length | 55.0^b^ | 17.5^b^ | .15 |  |  |  |  |  |  |  |  |  |  |  |  |  |  |  |  |  |  |  |  |  |  |  |  |  |  |
| 3. Meditation Sessions Weekly | 9.0^a^ | 7.0^a^ | .11 | .27 |  |  |  |  |  |  |  |  |  |  |  |  |  |  |  |  |  |  |  |  |  |  |  |  |  |
| 4. Meditation Hours Weekly | 7.5^a^ | 5.38^a^ | .06 | .71 ** | .81 ** |  |  |  |  |  |  |  |  |  |  |  |  |  |  |  |  |  |  |  |  |  |  |  |  |
| 5. Meditation Hours Total | 1560.0^a^ | 1794.0^a^ | .71 ** | .62 ** | .56 ** | .70 ** |  |  |  |  |  |  |  |  |  |  |  |  |  |  |  |  |  |  |  |  |  |  |  |
| 6. Meditation Retreats Total | 40.0^a^ | 55.0^a^ | .37* | .48 ** | .29 | .51 ** | .54 ** |  |  |  |  |  |  |  |  |  |  |  |  |  |  |  |  |  |  |  |  |  |  |
| 7. Meditation Retreats Yearly | 1.0^a^ | 1.0^a^ | .11 | .35 | .42* | .56 ** | .41* | .61 ** |  |  |  |  |  |  |  |  |  |  |  |  |  |  |  |  |  |  |  |  |  |
| 8. MAAS | 4.28 | 0.55 | .17 | -.15 | -.06 | -.12 | .02 | .11 | .34 |  |  |  |  |  |  |  |  |  |  |  |  |  |  |  |  |  |  |  |  |
| 9. TAS Difficulty Describing Feelings | 9.81 | 2.57 | -.20 | .19 | .15 | .14 | -.07 | -.19 | .07 | -.24 |  |  |  |  |  |  |  |  |  |  |  |  |  |  |  |  |  |  |  |
| 10. TAS Difficulty Identifying Feelings | 15.29 | 3.63 | .06 | .27 | .22 | .35 | .23 | .23 | .27 | -.29 | .46 ** |  |  |  |  |  |  |  |  |  |  |  |  |  |  |  |  |  |  |
| 11. TAS Total Score | 40.77 | 6.48 | .10 | .38* | .42* | .50 ** | .40* | .16 | .32 | -.26 | .74 ** | .78 ** |  |  |  |  |  |  |  |  |  |  |  |  |  |  |  |  |  |
| 12. MAIA-2 Noticing | 3.25^a^ | 0.38^a^ | .23 | .46 ** | .02 | .18 | .30 | .12 | .05 | .18 | -.06 | -.10 | .02 |  |  |  |  |  |  |  |  |  |  |  |  |  |  |  |  |
| 13. MAIA-2 Not-Distracting | 2.89 | 0.60 | -.17 | -.10 | -.16 | -.14 | -.26 | .05 | .08 | .50 ** | -.22 | -.29 | -.29 | .21 |  |  |  |  |  |  |  |  |  |  |  |  |  |  |  |
| 14. MAIA-2 Not-Worrying | 2.6^b^ | 0.6^b^ | -.02 | -.49 ** | -.14 | -.25 | -.22 | .14 | .27 | .55 ** | -.36* | -.24 | -.42* | -.25 | .34 |  |  |  |  |  |  |  |  |  |  |  |  |  |  |
| 15. MAIA-2 Attention Regulation | 2.97 | 0.44 | .10 | .30 | .23 | .30 | .25 | .16 | .28 | .51 ** | -.04 | .04 | .01 | .53 ** | .42* | .08 |  |  |  |  |  |  |  |  |  |  |  |  |  |
| 16. MAIA-2 Emotional Awareness | 3.32 | 0.44 | .30 | .13 | -.01 | -.01 | .19 | .24 | .09 | .05 | .03 | -.07 | -.03 | .50** | .32 | .02 | .38* |  |  |  |  |  |  |  |  |  |  |  |  |
| 17. MAIA-2 Self-Regulation | 3.03 | 0.38 | -.12 | .05 | -.09 | -.01 | -.09 | -.05 | .22 | .14 | .16 | .18 | .09 | .37* | .06 | -.07 | .38* | .29 |  |  |  |  |  |  |  |  |  |  |  |
| 18. MAIA-2 Body Listening | 2.89 | 0.42 | -.06 | .04 | -.14 | -.16 | -.16 | -.16 | -.28 | -.03 | .11 | -.08 | -.16 | .25 | .34 | -.11 | .37* | .45* | .08 |  |  |  |  |  |  |  |  |  |  |
| 19. MAIA-2 Trusting | 3.09 | 0.63 | -.02 | .00 | -.10 | -.14 | -.07 | -.19 | -.31 | -.21 | -.24 | -.38* | -.38* | .22 | .09 | -.04 | .23 | .37* | .05 | .49 ** |  |  |  |  |  |  |  |  |  |
| 20. ERQ Cognitive Reappraisal | 4.78 | 1.07 | .00 | -.18 | .02 | -.11 | -.06 | .04 | .15 | .07 | -.16 | -.29 | -.34 | -.21 | -.14 | .15 | -.12 | -.07 | .13 | -.25 | .06 |  |  |  |  |  |  |  |  |
| 21. ERQ Expressive Suppression | 2.53 | 0.82 | -.05 | .18 | .42* | .31 | .22 | -.07 | -.19 | -.15 | .12 | -.15 | .08 | .12 | -.11 | -.37* | .07 | .11 | .07 | .21 | -.02 | .02 |  |  |  |  |  |  |  |
| 22. PHQ-15 | 4.0^a^ | 3.57^a^ | .38* | .44* | .08 | .25 | .48 ** | .12 | .16 | -.20 | .22 | .13 | .31 | .17 | -.45* | -.44* | -.22 | -.06 | .00 | -.10 | -.28 | -.16 | .29 |  |  |  |  |  |  |
| 27. Criterion | 0.72 | 0.42 | .11 | -.28 | .00 | -.24 | -.12 | .09 | -.36* | -.26 | -.19 | -.14 | -.32 | -.04 | -.17 | .05 | -.09 | .13 | -.02 | .14 | .31 | .16 | .19 | -.12 |  |  |  |  |  |
| 28. Criterion during Light | 0.47 | 0.38 | .10 | -.40* | -.04 | -.30 | -.19 | .09 | -.28 | -.01 | -.31 | -.03 | -.29 | -.21 | -.11 | .21 | -.06 | -.17 | -.15 | -.12 | .04 | .22 | -.03 | -.19 | .83 ** |  |  |  |  |
| 29. Sensitivity | 1.98^b^ | 1.7^b^ | .01 | -.07 | .17 | .06 | -.07 | .39* | .06 | -.04 | .04 | .03 | -.09 | -.09 | .03 | .25 | .12 | .14 | -.03 | .09 | .03 | .14 | .10 | -.27 | .49  ** | .39* |  |  |  |
| 30. Sensitivity during Light | 2.47^b^ | 1.3^b^ | .02 | -.13 | .16 | .03 | -.06 | .39* | .04 | -.06 | .08 | .06 | -.06 | -.15 | .03 | .25 | .02 | .15 | -.08 | .04 | -.02 | .21 | .09 | -.30 | .40* | .35 | .91  ** |  |  |
| 31. Alpha Power Contralateral | 0.83^a^ | 1.37^a^ | -.09 | .04 | -.02 | .04 | -.08 | -.08 | -.02 | .11 | .02 | .11 | -.04 | -.05 | .22 | .34 | .13 | -.20 | -.31 | .36* | .07 | -.19 | -.16 | -.25 | -.25 | -.19 | .02 | .01 |  |
| 32. Alpha Power Contralateral Baselined | 0.02^a^ | 0.17^a^ | .03 | .18 | .36* | .29 | .21 | .03 | .19 | -.00 | .37* | .07 | .24 | -.13 | .03 | .08 | .12 | .22 | -.13 | .20 | -.07 | -.22 | .44* | .08 | -.19 | -.34 | .24 | .18 | .22 |

*M* and *SD* are used to represent the mean and standard deviation, respectively. Medians and interquartile ranges are shown for non-normally distributed data (marked with ^a^ for positive skew and ^b^ for negative skew). * indicates *p* < .05. ** indicates *p* < .01.

**Table A5.3**

**Within-group correlations across the non-meditator group (*n* = 33).** Means, standard deviations and Pearson correlation coefficients are shown. The variables in blue are non-normally distributed and were correlated with Spearman correlations. Medians and interquartile ranges are shown for non-normally distributed data (marked with ^a^ for positive skew and ^b^ for negative skew).

| Variable | *M* | *SD* | 1 | 2 | 3 | 4 | 5 | 6 | 7 | 8 | 9 | 10 | 11 | 12 | 13 | 14 | 15 | 16 | 17 | 18 | 19 | 20 |
| --- | --- | --- | --- | --- | --- | --- | --- | --- | --- | --- | --- | --- | --- | --- | --- | --- | --- | --- | --- | --- | --- | --- |
| 1. MAAS | 4.16 | 0.75 |  |  |  |  |  |  |  |  |  |  |  |  |  |  |  |  |  |  |  |  |
| 2. TAS Difficulty Describing Feelings | 11.76 | 3.44 | -.45** |  |  |  |  |  |  |  |  |  |  |  |  |  |  |  |  |  |  |  |
| 3. TAS Difficulty Identifying Feelings | 14.0^a^ | 9.0^a^ | -.65** | .51** |  |  |  |  |  |  |  |  |  |  |  |  |  |  |  |  |  |  |
| 4. TAS Total Score | 43.21 | 9.24 | -.49** | .82** | .84** |  |  |  |  |  |  |  |  |  |  |  |  |  |  |  |  |  |
| 5. MAIA-2 Noticing | 2.68 | 0.66 | .17 | -.34 | -.22 | -.15 |  |  |  |  |  |  |  |  |  |  |  |  |  |  |  |  |
| 6. MAIA-2 Not-Distracting | 1.80 | 0.82 | .40* | -.05 | -.23 | .02 | .20 |  |  |  |  |  |  |  |  |  |  |  |  |  |  |  |
| 7. MAIA-2 Not-Worrying | 2.18 | 0.56 | .16 | -.20 | -.33 | -.20 | .16 | .06 |  |  |  |  |  |  |  |  |  |  |  |  |  |  |
| 8. MAIA-2 Attention Regulation | 2.25 | 0.54 | .17 | -.40* | -.39* | -.27 | .55** | .07 | .45** |  |  |  |  |  |  |  |  |  |  |  |  |  |
| 9. MAIA-2 Emotional Awareness | 2.73 | 0.71 | -.12 | -.31 | -.18 | -.17 | .45** | .04 | .54** | .57** |  |  |  |  |  |  |  |  |  |  |  |  |
| 10. MAIA-2 Self-Regulation | 2.36 | 0.74 | .08 | -.31 | -.15 | -.29 | .38* | .14 | .45** | .55** | .57** |  |  |  |  |  |  |  |  |  |  |  |
| 11. MAIA-2 Body Listening | 2.33^b^ | 0.67^b^ | -.03 | -.05 | .14 | .10 | .28 | -.04 | .28 | .35* | .58** | .60** |  |  |  |  |  |  |  |  |  |  |
| 12. MAIA-2 Trusting | 2.69 | 0.82 | .26 | -.07 | -.25 | -.12 | .12 | .31 | .31 | .31 | .26 | .40* | .42* |  |  |  |  |  |  |  |  |  |
| 13. ERQ Cognitive Reappraisal | 5.17b | 1.17b | .00 | -.10 | -.08 | -.28 | -.09 | -.06 | .27 | .04 | .42* | .31 | .42* | .50** |  |  |  |  |  |  |  |  |
| 14. ERQ Expressive Suppression | 3.63 | 1.49 | -.55** | .57** | .44* | .59** | .02 | -.32 | -.10 | -.05 | .01 | -.05 | .02 | .06 | -.25 |  |  |  |  |  |  |  |
| 15. PHQ-15 | 3.21a | 4.93a | -.63** | .29 | .42* | .25 | -.32 | -.34 | -.37* | -.36* | -.10 | -.11 | -.17 | -.32 | .02 | .22 |  |  |  |  |  |  |
| 16. Criterion | 0.93 | 0.42 | .17 | .08 | -.19 | -.01 | .08 | .44* | .27 | .16 | .12 | .24 | -.11 | .31 | -.12 | -.02 | -.28 |  |  |  |  |  |
| 17. Criterion during Light | 0.67 | 0.48 | .15 | -.08 | -.03 | .01 | .22 | .23 | .22 | .34 | .20 | .39* | .09 | .25 | -.21 | -.11 | -.31 | .68** |  |  |  |  |
| 18. Sensitivity | 1.96 | 0.72 | -.32 | -.04 | .02 | .04 | .18 | -.16 | -.05 | .14 | .24 | .38* | -.02 | .07 | -.02 | .12 | .25 | -.23 | .04 |  |  |  |
| 19. Sensitivity during Light | 2.34 | 0.81 | -.17 | -.04 | -.00 | -.04 | .17 | -.20 | -.18 | .02 | .10 | .26 | .04 | .19 | -.06 | .08 | .28 | -.28 | .02 | .87** |  |  |
| 20. Alpha Power Contralateral | 2.26a | 4.24a | -.11 | -.00 | .15 | .13 | .20 | .02 | .29 | .07 | .21 | .04 | .10 | .28 | .21 | .21 | -.22 | .02 | .17 | -.09 | -.13 |  |
| 21. Alpha Power Contralateral Baselined | 0.20 | 0.27 | -.25 | .27 | .27 | .22 | -.24 | .03 | -.17 | -.16 | -.06 | -.19 | .27 | -.04 | -.06 | .16 | .00 | -.15 | -.16 | -.16 | -.08 | .34 |

*M* and *SD* are used to represent the mean and standard deviation, respectively. Medians and interquartile ranges are shown for non-normally distributed data (marked with ^a^ for positive skew and ^b^ for negative skew). * indicates *p* < .05. ** indicates *p* < .01.
